# Saarvienin A—A Novel Glycopeptide with Potent Activity against Drug‐Resistant Bacteria

**DOI:** 10.1002/anie.202425588

**Published:** 2025-04-25

**Authors:** Amninder Kaur, Jaime Felipe Guerrero‐Garzón, Sari Rasheed, Martin Zehl, Franziska Fries, Bernd Morgenstern, Sergey B. Zotchev, Rolf Müller

**Affiliations:** ^1^ Helmholtz Institute for Pharmaceutical Research Saarland (HIPS) Helmholtz Centre for Infection Research (HZI) PharmaScienceHub (PSH) Saarland University Campus 66123 Saarbrücken Germany; ^2^ German Centre for Infection Research (DZIF) Partner Site Hannover‐Braunschweig 38124 Braunschweig Germany; ^3^ Department of Pharmaceutical Sciences Division of Pharmacognosy University of Vienna Vienna 1090 Austria; ^4^ Department of Analytical Chemistry Faculty of Chemistry University of Vienna Vienna 1090 Austria; ^5^ Department of Chemistry Saarland University 66123 Saarbrücken Germany; ^6^ Department of Pharmacy Saarland University 66123 Saarbrücken Germany

**Keywords:** *Amycolatopsis* sp, Glycopeptide antibiotic, Gram‐positive bacteria, Structure elucidation, Vancomycin‐resistance

## Abstract

A member of a new family of glycopeptides, named saarvienin A, was isolated from a rare actinomycete *Amycolatopsis* sp. YIM10. Extensive NMR and MS analyses revealed a halogenated peptide core comprising four amino acids cyclized via a ureido linkage with an exocyclic 2‐hydroxy‐3‐(4‐hydroxyphenyl)propyl residue connected to a five‐sugar/aminosugar chain. Two of the three aminosugars constitute the *N*‐methylated and *N*,*O*‐dimethylated derivatives of eremosamine (4‐*epi*‐vancosamine) that have not been reported in any natural product. Saarvienin A exhibits potent activity against a range of Gram‐positive bacteria, effectively overcoming resistance to several frontline antibiotics in clinical isolates. It demonstrates an eight‐fold reduction in minimum inhibitory concentrations (MICs) against methicillin‐resistant, vancomycin‐intermediate, and daptomycin‐resistant *Staphylococcus aureus* compared to vancomycin.

## Graphical abstract

A structurally distinct glycopeptide, saarvienin A, represents a new family of glycopeptide antibiotics consisting of five sugar/aminosugar units connected to a halogenated peptide core. Saarvienin A overcomes vancomycin resistance and shows strong activity against drug‐resistant Gram‐positive bacteria, in particular, high‐priority pathogens such as methicillin‐resistant, vancomycin‐intermediate, and daptomycin‐resistant *Staphylococcus aureus*.

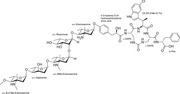

## Introduction

Glycopeptide antibiotics (GPAs) constitute compounds of microbial origin that serve as last‐resort treatments for severe multidrug‐resistant Gram‐positive bacterial infections.^[^ ^1^, ^2^ ^]^ Some important members of this compound class, such as the first‐ and second‐generation (semi‐synthetic) GPAs vancomycin, teicoplanin, dalbavancin, oritavancin, and telavancin, are available as marketed drugs.^[^ ^3^ ^]^ They comprise unique cyclic peptide cores that are typically glycosylated and may contain additional lipophilic fatty acid chains. GPAs have been divided into five distinct structural types (I–V) depending on the presence of aliphatic or aromatic chains in amino acids 1 and 3 of the polypeptide, aliphatic side chain on sugar units, extra ring systems, or tryptophan linkage to the central amino acid.^[^ ^4^ ^]^ The mode of action of GPAs involves binding to the terminal d‐alanyl‐d‐alanine moiety of Lipid II, an intermediate in peptidoglycan layer synthesis, through five hydrogen bonds.^[^ ^5^, ^6^ ^]^ This binding blocks the peptidoglycan maturation, compromising the integrity of the cell envelope and leading to cell death due to osmotic stress.^[^ ^5^ ^]^ It was initially thought that resistance to GPAs would be difficult to develop due to their unique target, which would necessitate coordinated changes in multiple enzymes within the peptidoglycan synthesis pathway to circumvent inhibition. However, GPA resistance eventually emerged, posing a severe threat and raising serious concerns within health care systems globally.^[^ ^7^ ^]^ Resistance to GPAs is achieved by a remarkable and rather simple variation of the target,^[^ ^6^ ^]^ where GPA‐resistant cells replace the d‐alanyl‐d‐alanine moiety with d‐alanyl‐d‐lactate or d‐alanyl‐d‐serine, thereby significantly reducing antibiotic affinity.^[^ ^1^, ^6^ ^]^ Although extensive efforts by medicinal chemists have yielded promising variations of traditional GPAs,^[^ ^8^, ^9^, ^10^ ^]^ the emerging resistance necessitates the continued discovery of structurally distinct GPAs capable of exhibiting novel, selective, and potent biological activities.

**Figure 1:**
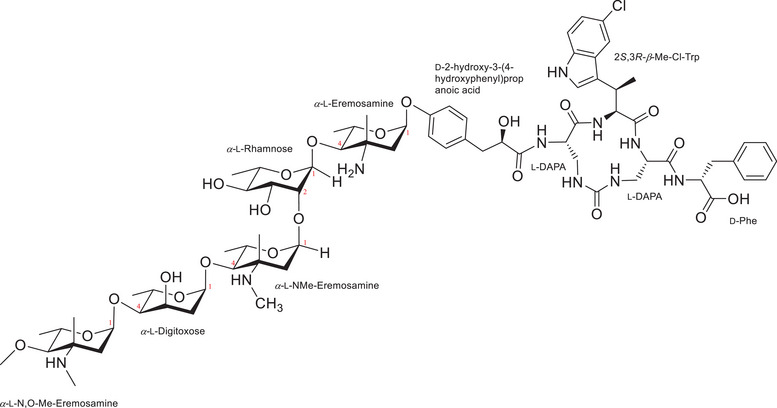
Chemical structure of saarvienin A (**1**).

The genus *Amycolatopsis* is a historically important genus in the phylum Actinomycetota (Actinobacteria) that is well‐known for producing antibiotics such as vancomycin, rifamycin, and chelocardin.^[^ ^11^ ^]^ *Amycolatopsis* sp. YIM10 that was isolated from a Chinese rare earth mine has proved to be a rich reservoir of biosynthetic gene clusters (BGCs).^[^ ^12^ ^]^ However, only a few secondary metabolites have been identified either in its fermentation broths or via the genome mining approach, and none of them have shown strong antimicrobial activity.^[^ ^12^ ^]^ Still, we observed significant antimicrobial effects of some culture extracts. Further fractionation of these extracts led to the isolation and identification of a new glycopeptide, named saarvienin A (**1**; Figure 1). Extensive NMR and MS analyses revealed a pentasugar/aminosugar chain connected to a halogenated peptide core, where three of the four amino acids form a macrocycle via a urea‐type carbonyl linkage. A Scifinder search conducted to find the closest structurally similar compounds yielded structurally distinct vancomycin derivatives as the closest hits, indicating that the chemical structure of **1** represents a new family of potent glycopeptides capable of overcoming vancomycin resistance while simultaneously exhibiting selective biological activity against Gram‐positive bacteria. In this study, we report the identification, purification, structure elucidation, and comprehensive microbiological in vitro testing of the first representative of a new GPA family.

## Results and Discussion

Bioactivity‐guided fractionation of the fermentation extracts of *Amycolatopsis* sp. YIM10 identified fractions that showed activity against two Gram‐positive bacteria, *Staphylococcus carnosus* and *Bacillus subtilis*. Dominant compound (**1**) in the bioactive fractions was assigned the sum formula C_73_H_105_ClN_10_O_22_ (unsaturation number = 26) based on the accurate mass and isotopic pattern of the predominant doubly (*m/z* 755.3619 [M + 2H]^2+^) and triply (*m/z* 503.9106 [M + 3H]^3+^) charged ions and their fragment ions (Figures S1–S3). Collision‐induced dissociation yielded a fragmentation pattern consistent with a stabilized, halogenated peptide moiety with the sum formula C_37_H_40_ClN_7_O_9_ and an oligosaccharide moiety with the sum formula C_36_H_65_N_3_O_13_, likely constituting three different amino sugars, one deoxy sugar, and one dideoxy sugar. Based on these results, it was initially hypothesized that this so far undescribed compound could be related to the vancomycin‐like family of glycopeptides. Preliminary attempts of purification using affinity chromatography revealed that unlike vancomycin, the novel compound does not bind to the d‐alanyl‐d‐alanine peptide motif, suggesting a different mode of action. Compound **1** was eventually purified using preparative reverse‐phase HPLC, and a combination of HRMSMS, 1D/2D NMR, X‐ray diffraction (XRD), and chemical derivatization techniques was used to determine its complete structure, which was found to be significantly different from that of vancomycin or its derivatives.

**Figure 2:**
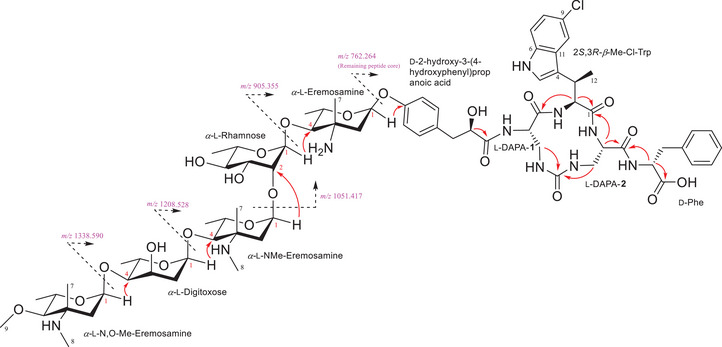
Key HMBC correlations (→) and HRMSMS data used to establish the connectivity of various units in saarvienin A (**1**).

The best solvent to acquire the NMR data was found to be CD_3_OD:D_2_O (∼3:1), in which sharp and mostly well‐resolved signals were observed. However, the measured NMR shifts varied slightly depending on the concentration of **1** and the CD_3_OD:D_2_O ratio in the NMR samples. Although NMR data were collected in CD_3_OD/D_2_O mixture and DMSO‐*d*_6_ (298 and 313 K), only the data relevant to the discussion are included herein. HRESIMSMS data of **1** showed sequential losses of five sugar/amino sugar type units (Figures 2 and S3), corresponding to the losses of C_9_H_17_NO_2_ (*m/z* 1338.590 [M + H]^+^), C_9_H_17_NO_2_‐C_6_H_10_O_3_ (*m/z* 1208.528 [M + H]^+^), C_9_H_17_NO_2_‐C_6_H_10_O_3_‐C_8_H_15_NO_2_ (*m/z* 1051.417 [M + H]^+^), C_9_H_17_NO_2_‐C_6_H_10_O_3_‐C_8_H_15_NO_2_‐C_6_H_10_O_4_ (*m/z* 905.355 [M + H]^+^), and C_9_H_17_NO_2_‐C_6_H_10_O_3_‐C_8_H_15_NO_2_‐C_6_H_10_O_4_‐C_7_H_13_NO_2_ (*m/z* 762.264 [M + H]^+^) segments. Analogous sugar‐type signals were also observed in the ^1^H and ^13^C NMR data (Figures S4–S7, Table S1), with five characteristic anomeric proton signals observed at 4.9–5.6 ppm and 90–105 ppm in the ^1^H and ^13^C NMR spectra, respectively.

Detailed analysis of 1D and 2D NMR data (Figures S4–S18, Table S1) allowed the identification of the C_7_H_13_NO_2_ unit as 4‐*epi*‐vancosamine, also known as eremosamine (Ere).^[^ ^13^ ^]^ Two individual spin systems connecting H_Ere_‐1 (*δ*_H_ 5.60) to H_Ere_‐2_a/b_ (*δ*_H_ 2.28/2.20; CH_2_) and H_Ere_‐4 (*δ*_H_ 3.51) to H_Ere_‐5 (*δ*_H_ 3.88) and H_Ere_‐6 (*δ*_H_ 1.34, CH_3_) were identified using TOCSY and COSY correlations. HMBC correlations from the methyl group at *δ*_H_ 1.59 (H_Ere_‐7) to C_Ere_‐2 (*δ*_C_ 39.99), C_Ere_‐3 (*δ*_C_ 57.1), and C_Ere_‐4 (*δ*_C_ 86.8) connected the two spin systems via a quaternary carbon (C_Ere_‐3). The ^13^C NMR shift of C_Ere_‐3 was in line with the placement of the amine group at this carbon. ROESY correlations and proton‐proton coupling constants (*J*_HH_) were used to determine its relative configuration (Figure S19). A lack of a large coupling constant for the anomeric proton signal (H_Ere_‐1, *δ*_H_ 5.60 (br d, 3.1)) was consistent with the presence of the α‐anomer of Ere, while a large coupling constant (8.9 Hz) observed for H_Ere_‐4 and H_Ere_‐5 placed these in diaxial orientation to each other. ROESY correlations observed between H_Ere_‐5, H_Ere_‐7, and H_Ere_‐2a allowed their placement on the same face of the ring system. Further analysis of the NMR data identified two additional aminosugars with spin systems and connectivities similar to those in Ere. These units, corresponding to the building blocks with sum formulae C_8_H_15_NO_2_ and C_9_H_17_NO_2_ observed in HRESIMSMS data, were determined to be *N*‐methylated (NMe‐Ere) and *N*,*O*‐dimethylated (N,O‐Me‐Ere) derivatives of Ere, respectively, with N,O‐Me‐Ere being one of the terminal sugar moiety. HMBC correlation from the methyl protons at *δ*_H_ 2.64 (H_NMe‐Ere_‐8) to the quaternary carbon at *δ*_C_ 61.95 (C_NMe‐Ere_‐3) was consistent with the presence of an *N*‐Me group. Similarly, HMBC correlations from the methyl group at *δ*_H_ 2.60 (H_N,O‐Me‐Ere_‐8) to *δ*_C_ 61.86 (C_N,O‐Me‐Ere_‐3), as well as those from OMe (*δ*_H_ 3.58, H_N,O‐Me‐Ere_‐9) to the methine carbon at *δ*_C_ 86.0 (C_N,O‐Me‐Ere_‐4), located their positions in N,O‐Me‐Ere. Furthermore, key ROESY correlations (Figure S19) and *J*_HH_ values were analogous to those in Ere, indicating that both aminosugars were also α‐anomers. Notably, both N‐Me‐Ere and N,O‐Me‐Ere have not been reported in any natural products described in the literature.

The remaining two sugars were determined to be rhamnose (Rha) and digitoxose (Dig).^[^ ^14^ ^]^ TOCSY and COSY correlations were used to readily identify the complete spin systems present in these sugars (Figures S8, S9). Anomeric proton H_Dig_‐1 (*δ*_H_ 5.02) exhibited a broad singlet, indicating the α‐anomeric diastereomer of Dig. H_Dig_‐4 (*δ*_H_ 3.40) and H_Dig_‐5 (*δ*_H_ 4.29) were mutually coupled to each other via a coupling constant of 8.6 Hz, positioning them axial to each other, while ROESY correlation between H_Dig_‐3 (*δ*_H_ 4.32) and H_Dig_‐4 placed these on the same face of the ring system, that is, H_Dig_‐3 in equatorial orientation. In Rha, a coupling constant of 9.4 Hz observed for H_Rha_‐3 (*δ*_H_ 3.83), H_Rha_‐4 (*δ*_H_ 3.37), and H_Rha_‐5 (*δ*_H_ 3.65) indicated their axial alignment to each other, whereas a small *J* value (2.3 Hz) for H_Rha_‐2 (*δ*_H_ 3.93) was consistent with its equatorial orientation. As *J* values could not be used to determine the alpha or beta form of Rha, ROESY correlations were employed. While ROESY correlations were observed between *trans*‐diaxially placed H_Rha_‐3 and H_Rha_‐5, a lack of such correlation between H_Rha_‐1 (*δ*_H_ 4.96) and H_Rha_‐3/5 indicated that Rha was also present in the α‐anomeric form.^[^ ^15^ ^]^ Mass differences between fragment ions corresponding to C_6_H_10_O_3_ and C_6_H_10_O_4_ also supported the presence of Rha and Dig.

Establishing the connectivity of the sugar units in series to each other was relatively straightforward owing to the key HMBC and ROESY correlations observed in the NMR data (Figures S11, S12), as well as sequential losses observed in HRESIMSMS data (Figure S3). HMBC correlations from anomeric protons H_N,O‐Me‐Ere_‐1 (*δ*_H_ 5.17) to C_Dig_‐4 (*δ*_C_ 77.9), H_Dig_‐1 (*δ*_H_ 5.02) to C_NMe‐Ere_‐4 (*δ*_C_ 80.2), H_NMe‐Ere_‐1 (*δ*_H_ 5.14) to C_Rha_‐2 (*δ*_C_ 81.4), and H_Rha_‐1 (*δ*_H_ 4.96) to C_Ere_‐4 (*δ*_C_ 86.8), as well as the corresponding ROESY correlations, established the connectivity to the sugar units as N,O‐Me‐Ere (1→4) Dig (1→4) NMe‐Ere (1→2) Rha (1→4) Ere (Figures 2 and S22).

The molecular formula corresponding to the fragment ion at *m/z* 762.264 was determined to be C_37_H_41_ClN_7_O_9_^+^, suggesting a peptide core comprising five to seven amino acids. This and several other related MS signals showed a distinct isotope pattern consistent with the presence of one Cl atom on one of the amino acid units. Aromatic ^1^H NMR signals were observed for 13 protons, together with the analogous ^13^C NMR signals, four of the latter corresponding to 2 equivalent protons each, consistent with the presence of phenylalanine (Phe) or tyrosine (Tyr) type units. Comprehensive analysis of these data indicated the presence of one Phe, one β‐methyl‐9‐chlorotryptophan (β‐Me‐Cl‐Trp), and one Tyr‐type unit, identified as 2‐hydroxy‐3‐(4‐hydroxyphenyl)propanoic acid (HHPP). Notably, the ^13^C chemical shift of C_HHPP_‐2 (*δ*_C_ 73.7) was diagnostic of an oxygenation at this position, indicating that this unit was HHPP and not Tyr. The presence of HHPP was also confirmed via Marfey's analysis. An HMBC correlation from H_Ere_‐1 (*δ*_H_ 5.60) to C_HHPP_‐7 (*δ*_C_ 156.6), as well as a strong ROESY correlation between H_Ere_‐1 and H_HHPP_‐6/8 (*δ*_H_ 6.94), showed attachment of the sugar chain to the peptide core via C_HHPP_‐7. While most of the Phe NMR resonances were consistent with those expected, the ^1^H NMR signals for H_Phe_‐5/9 (*δ*_H_ 6.61) were unusually upfield‐shifted, likely due to the shielding effect of the nearby aromatic ring systems. In β‐Me‐Cl‐Trp, HMBC correlations from the methyl group (H_Trp_‐12, *δ*_H_ 1.28) to C_Trp_‐2 (*δ*_C_ 60.1), C_Trp_‐3 (*δ*_C_ 33.2), and C_Trp_‐4 (*δ*_C_ 118.1) located its beta‐position in this unit. Position of the chlorine at C_Trp_‐9 (*δ*_C_ 125.6) was determined based on HMBC NMR analysis of the ring system. Unexpectedly, one of the flash chromatography fractions was found to contain significant amounts of β‐methyl‐9‐chlorotryprophan as an individual component, which was purified and subjected to complete characterization using NMR and optical rotation studies. Fortunately, a crystal of this compound was also obtained in methanol, and its absolute configuration was determined as (2*S*,3*R*)‐β‐Me‐Cl‐Trp (Figure S20). This individual compound was later used as a standard to determine the absolute configuration of the β‐Me‐Cl‐Trp unit present in **1**.

With most of the units accounted for, only two sets of very similar signals remained to be annotated in the NMR data. These constituted two pairs of diastereotopic protons at *δ*_H_ 2.94/3.79 (2H) and *δ*_H_ 3.00/3.54 (2H) coupled to two methine protons at *δ*_H_ 4.33 and *δ*_H_ 4.25, respectively. After considering several possibilities and talking into account the molecular formula as well as remaining signals in the ^13^C NMR data, these units were finally assigned to diaminopropionic acid (DAPA), the presence of which was also confirmed via Marfey's analysis. Another important observation was made when NMR data were collected in DMSO‐*d*_6_. Although the signal shape and resolution were found to be relatively deteriorated in DMSO‐*d*_6_ in comparison to those in CD_3_OD/D_2_O mixture, TOCSY data (Figure S16) showed a four‐proton spin system for the HHPP unit, indicating the presence of a free hydroxyl group in HHPP. Additionally, two five‐proton spin systems were observed for the two DAPA units. Based on the TOCSY data collected at two different D9 values (0.09 and 0.03; Figure S17), one of the NH protons of each DAPA unit was attached to the alpha position and the other to the beta position, suggesting the absence of a free amine in DAPA, and hence, extension of the amino acid chain via both amine groups.

Establishing the connectivity of the peptide core proved to be somewhat challenging due to a lack of key HMBC correlations observed to the carbonyl carbons. Therefore, HMBC data were collected for samples containing different concentrations of **1** at several different CNST13 values (Figures S13, S14). HMBC correlations from H_Phe_‐2 (*δ*_H_ 4.35) to C_Phe_‐1 (*δ*_C_ 177.6) and C_DAPA‐2_–1 (*δ*_C_ 170.2), H_DAPA‐2_–2 (*δ*_H_ 4.25) to C_DAPA‐2_–1 and C_Trp_‐1 (*δ*_C_ 174.0), and H_Trp_‐2 (*δ*_H_ 4.84) to C_Trp_‐1 and C_DAPA‐1_–1 (*δ*_C_ 172.3) established the presence of the DAPA‐1—β‐Me‐Cl‐Trp—DAPA‐2—Phe chain. Furthermore, H_DAPA‐1_–3a (*δ*_H_ 2.94) and H_DAPA‐2_–3a (*δ*_H_ 3.00) showed HMBC correlations to the only remaining unannotated carbonyl carbon at *δ*_C_ 161.0, whose chemical shift was indicative of a urea‐type carbonyl. Connectivity of the two DAPA side chains via a ureido carbonyl to form a macrocycle, and of the HHPP unit to DAPA‐1 via an amide bond to the alpha‐NH was consistent with the molecular formula and unsaturation number of **1**. NOESY correlations between the beta‐NH protons of the two DAPA units in DMSO‐*d*_6_ also supported the connection of DAPA units via the ureido carbonyl (Figure S18). Moreover, the masses and molecular formulae of the fragments obtained upon partial acidic hydrolysis (6 N HCl, 110 °C, 15–20 min) of **1** were consistent with the structure of the proposed peptide core (Figure S23). Loss of the *C*‐terminal Phe and the *N*‐terminal HHPP from the peptide core to afford the three‐amino‐acid cyclic ring system C_19_H_23_ClN_6_O_5_ (*m/z* 451.149 [M + H]^+^) connected via a ureido linkage provided additional support for this part.

Absolute configuration of the amino acid residues was determined using Marfey's analysis (Table S2, Figure S24). After acidic hydrolysis (6 N HCl, 110 °C, 18–24 h) of **1**, the hydrolysate and corresponding reference amino acids were subjected to derivatization with 1‐fluoro‐2,4‐dinitrophenyl‐5‐leucine‐amide (l‐FDLA or d‐FDLA; Marfey's reagent) followed by LCMS analysis. Subsequent comparison of the retention times and MS data of the derivatized amino acids in the hydrolysate to those of the reference standard amino acids (Table S2, Figure S24) indicated the presence of d‐HHPP, d‐Phe, and both d‐ and l‐DAPA. As DAPA is known to racemize during acidic hydrolysis, **1** was also hydrolyzed using DCl/D_2_O (20 wt% DCl in D_2_O) and then derivatized using Marfey's reagents.^[^ ^16^ ^]^ Presence of only the first isotope ion for d‐DAPA and a mixture of monoisotopic and first isotope ions for l‐DAPA residues (Figure S26) suggested that d‐DAPA was formed from l‐DAPA via racemization upon hydrolysis in 6 N HCl.^[^ ^16^ ^]^ Therefore, both DAPA units were assigned l‐configuration. β‐Me‐Cl‐Trp was found to degrade during acidic hydrolysis, and therefore, **1** was also hydrolyzed under basic conditions (5 N NaOH, 110 °C, 24 h) and then subjected to Marfey's analysis, where the isolated (2*S*,3*R*)‐β‐Me‐Cl‐Trp was used as a reference standard. As expected, some racemization was observed under basic conditions generating the other two possible diastereomers as minor peaks, that is, (2*R*,3*R)*‐β‐Me‐Cl‐Trp‐ l‐DLA (21.5 min) and (2*R*,3*R)*‐β‐Me‐Cl‐Trp‐d‐DLA (19.2 min) in addition to major peaks observed for the expected (2*S*,3*R*)‐β‐Me‐Cl‐Trp‐l‐DLA (19.6 min) and (2*S*,3*R*)‐β‐Me‐Cl‐Trp‐d‐DLA (21.3 min) derivatives. However, the β‐Me‐Cl‐Trp unit in **1** could be readily assigned as (2*S*,3*R*)‐β‐Me‐Cl‐Trp as the retention times of the major peaks observed for its l‐DLA and d‐DLA derivatives matched well with those of the corresponding Marfey's derivatives of the isolated standard (Figure S25), thereby ruling out other stereochemical possibilities.

To determine the absolute configuration of the sugar units, different derivatization methods were used. The natural product eremomycin was purchased and hydrolyzed (1 N HCl, 60 °C, 4 h) to obtain Ere standard, which was further subjected to butanolysis using *S/R*‐2‐butanol. l‐Rha and d‐Dig standards were directly subjected to butanolysis reaction. Saarvienin A was similarly hydrolyzed and derivatized, and this mixture, as well as the derivatized standards, were analyzed using LCMS. A comparison of the retention times and MS data allowed the identification of l‐Ere and l‐Rha (Table S3, Figures S27–S28). As NMe‐Ere and N,O‐Me‐Ere are simply the methylated derivatives of Ere and no new stereocenters are generated by these substituents, these are assigned an analogous absolute configuration to Ere. However, butyl derivatives of Dig could not be separated even after using different columns and method optimization strategies. GCMS analysis after silylation was also attempted, but was unsuccessful. To facilitate the separation of Dig derivatives, we decided to use a bulkier derivatization agent. As Mosher's reagent [*S/R*‐α‐methoxy‐α‐(trifluoromethyl)phenylacetyl chloride (MTPACl)] was readily available in our laboratory, d‐Dig and l‐Rha standards were individually treated with an excess of *S/R*‐MTPACl in pyridine and directly subjected to LCMS analysis. After hydrolysis, compound **1** was also derivatized in a similar manner. Comparison of the retention times and MS data of the derivatized sugars in the hydrolysate to those of the derivatized standards was consistent with the presence of l‐Dig and l‐Rha (Table S4, Figures S29–S30), thereby completing the absolute configuration assignment of all individual units in **1**.

The in vitro antibacterial activity of **1** was assessed against a panel of Gram‐positive and Gram‐negative pathogens (Table 1). The compound demonstrated notable efficacy against Gram‐positive bacterial pathogens, including vancomycin‐resistant enterococci (VRE) and methicillin‐resistant, vancomycin‐intermediate resistant (MRSA/VISA) and daptomycin‐resistant *S. aureus* (DRSA). Compound **1** exhibited a minimum inhibitory concentration (MIC) of 1 µg mL^−1^ against vancomycin‐resistant *Enterococcus* *faecalis* (DSM12956, vanB‐positive) and *Enterococcus* *faecium* (DSM17050, vanA‐positive), significantly outperforming vancomycin, which had MIC values of 16 µg mL^−1^ and >64 µg mL^−1^, respectively. The compound also showed a low MIC of 0.5 µg mL^−1^ against MRSA/VISA and effectively overcame resistance in a highly DRSA strain (HG001)^[^ ^17^ ^]^ with an MIC of 1 µg mL^−1^. Additionally, **1** displayed activity against the non‐pathogenic *Mycobacterium smegmatis* (MIC 1 µg mL^−1^) but demonstrated limited efficacy against *Mycobacterium tuberculosis* (*Mtb* H37Ra, MIC 32 µg mL^−1^). In contrast, **1** lacked activity against Gram‐negative pathogens (MIC >64 µg mL^−1^), including *Klebsiella pneumoniae*, *Escherichia coli*, *Salmonella enterica*, and *Pseudomonas aeruginosa*.

**Table 1:** Minimum inhibitory concentrations (MICs) of saarvienin A against bacterial strains.

	MIC (µg mL^−1^)	
	Saarvienin A	Vancomycin
*B. subtilis* DSM 10	0.125	0.25
*E. faecalis* DSM 12956 (VRE, vanB positive)	1	16
*E. faecalis* ATCC 29212	2	4
*E. faecium* DSM 17050 (VRE, vanA positive)	1	>64
*S. aureus* ATCC 29213	0.25	2
*S. aureus* N315 (MRSA)	0.5	2
*S. aureus* Mu50 (MRSA/VISA)	1	8
*S. aureus* DSM 11822 (MDR)	0.5	1
*S. aureus* HG001‐DRSA^a)^	1	4
*S. epidermidis* DSM 28765	0.25	4

*M. smegmatis* mc^2^155	1	2
*M. tuberculosis* H37Ra ATCC 25177	32	64

*K. pneumoniae* DSM 681	>64	>64
*S. enterica* DSM5569	>64	>64
*E. coli* ATCC 25922	>64	>64
*E. coli* K12 Δ*tolC*	64	>64
*E. coli* K12 Δ*tolC* *(tolC‐deficient)* + PMBN	32	>64
*E. coli* WO153 (AB1157*, recJ asmB1*, Δ*tolC*)	2	32
*E. coli* K12 Δ*acrB*	>64	>64
*P. aeruginosa* PA14	>64	>64
*P. aeruginosa* PA14 Δ*mexAB*	>64	>64

MIC values are expressed in µg mL^−1^ and represent the mean of three independent biological replicates, each performed in technical triplicate.

^a)^ MIC of daptomycin: 32 µg mL^−1^.

To investigate whether the lack of activity of **1** against Gram‐negative pathogens is influenced by efflux and/or reduced membrane diffusion, bioactivity testing was conducted using efflux‐deficient and hyperpermeable *E. coli* strains. Efflux via the major AcrAB‐TolC pump appeared to play a minor role, as *E. coli* ΔacrB remained non‐susceptible to **1** and deletion of the outer membrane TolC component only led to low activity (MIC 64 µg mL^−1^). However, the activity of **1** was slightly enhanced when the *E. coli* ΔtolC strain was co‐incubated with sub‐lethal concentrations of polymyxin B nonapeptide (PMBN), increasing outer membrane permeability and reducing the MIC to 32 µg mL^−1^. Interestingly, the compound displayed potent activity (MIC 2 µg mL^−1^) against *E. coli* WO153, which carries mutations in the *asmB1* allele of *lpxC* (involved in lipid A biosynthesis) and a deletion of the *tolC* gene,^[^ ^18^, ^19^ ^]^ resulting in enhanced permeability and reduced efflux. These findings demonstrate that the antibacterial target of **1** is also present in *E. coli* and that the resistance of Gram‐negative strains is likely due to its inability to efficiently cross the outer membrane barrier.

Building on the promising activity of saarvienin A in overcoming resistance to clinically relevant antibiotics, we investigated its MIC against a panel of VRE clinical isolates with reduced susceptibility to daptomycin. Saarvienin A demonstrated potent activity, achieving an MIC50/90 of 0.5 µg mL^−1^ across the 26 tested isolates, effectively overcoming resistance to vancomycin, teicoplanin, ciprofloxacin, ampicillin, and gentamicin (Tables 2 and S5).

**Table 2:** Minimum inhibitory concentrations (MICs) of saarvienin A against a collection of vancomycin‐resistant *E. faecium* clinical isolates with reduced susceptibility to daptomycin (*n* = 26).

	MIC (µg mL^−1^)		
	MIC_50_	MIC_90_	Range
Saarvienin A	0.5	0.5	0.5–1
Daptomycin	8	8	8–16
Vancomycin	>64	>64	64 to >64
Linezolid	4	4	2–4
Teicoplanin	64	32	2 to >64
Ciprofloxacin	>64	64	8 to >64
Ampicillin	>64	>64	64 to >64
Gentamicin	>64	>64	64 to >64

Values represent the mean of two independent experiments.

Cytotoxicity of **1** was assessed using the HepG2 (human hepatocellular carcinoma) cell line, revealing toxic effects with an IC50 of 13 µg mL^−1^ leaving some potential application window for **1**. However, these results indicate that despite its promising antibacterial activity, further structural optimization and evaluation are needed to enhance its selectivity and reduce cytotoxicity.

The chemical structure of **1** provided some clues regarding which enzymes must be involved in its biosynthesis. In particular, the peptide core is presumably assembled by a modular non‐ribosomal peptide synthetase (NRPS), the tryptophan moiety of the core must be modified by a halogenase and a methyltransferase, and five deoxy(amino)sugar residues must be tethered to the peptide core by several glycosyltransferases. In addition, the enzymes responsible for the biosynthesis of DAPA may be involved. With this in mind, we analyzed the BGCs of *Amycolatopsis* sp. YIM10 previously detected in its genome.^[^ ^12^ ^]^ BGC14 was the only likely candidate that fulfilled the abovementioned criteria relatively well (Table S6). This gene cluster contains two genes, ctg2_4530 and ctg2_4531 encoding four‐ and two‐modular NRPS proteins, respectively, suggesting them to generate a six‐amino acid peptide core. However, in one of the condensation domains in the NRPS encoded by ctg_4531, the first His residue in the conserved active site motif HHxxDG is replaced with Cys. The latter suggests that the condensation step supposed to be catalyzed by this domain may be skipped, which is known to be the case in certain NRPS systems.^[^ ^20^ ^]^ Module skipping may therefore explain why the six‐modular NRPS can assemble the 5‐amino acid peptide core of **1**. BGC14 also contains transcriptionally/translationally coupled genes for a tryptophan halogenase and methyltransferase, seven genes presumably encoding enzymes for the biosynthesis of eremosamine and its *N*‐ and *O*‐methylation, as well as four genes for glycosyltransferases likely to be responsible for the glycosylation of the peptide core with five sugar residues (Table S6). Most likely, the genes involved in the biosynthesis of rhamnose and digitoxose sugar units are located outside of BGC14. Two genes encoding enzymes known to be involved in the biosynthesis of DAPA are also found within the cluster along with two genes for an aromatic amino acid lyase and an AMP‐binding protein that may be involved in the biosynthesis and activation of HHPP, possibly following parts of the pathway for phloretic acid biosynthesis.^[^ ^21^ ^]^ However, extensive genetic and biochemical studies including attempts to clone this cluster, its mutational analysis, and biochemical characterization of the encoded enzymes are needed and currently under way to validate these suppositions.

## Conclusion

Saarvienin A, representing the first member of a new family of GPAs, was discovered in the fermentation extracts of *Amycolatopsis* sp., a genus that continues to be a prolific producer of new antibiotics. The complete structure of **1**, including its relative and absolute configuration, was determined using extensive NMR, MSMS, XRD, and chemical derivatization methods. In comparison to vancomycin, **1** showed significantly enhanced activity against Gram‐positive bacteria, including high‐priority pathogens such as VRE and MRSA/VISA, and DRSA. This activity was further demonstrated on clinical isolates, where saarvienin A displayed remarkable potency, effectively overcoming resistance to clinically relevant antibiotics. Since the compound exhibits cytotoxic activity against eukaryotic cell line, additional optimization is required to enhance its potency and selectivity. Future research efforts should aim at modification of its chemical structure to improve its potential as a novel clinically significant antibacterial agent.

## Supporting Information

Instrumentation details, experimental methodology, and NMR, MS, XRD, and bioactivity data are provided in the supporting information. The authors have cited additional references within the Supporting Information.^[^ ^12^, ^22^, ^23^, ^24^, ^25^, ^26^, ^27^, ^28^ ^]^

## Conflict of Interests

The authors declare no conflict of interest.

## Supporting information



Supporting Information

## Data Availability

The data that support the findings of this study are available in the supplementary material of this article.
