# Supplementary material for: Saarvienin A—A Novel Glycopeptide with Potent Activity against Drug‐Resistant Bacteria
Source: Angew Chem Int Ed Engl. 2025 Apr 25;64(25):e202425588. doi: 10.1002/anie.202425588 (PMC12171682; doi:10.1002/anie.202425588)
Supplement: Supplementary file 1 — Supporting Information [file ANIE-64-e202425588-s001.docx]

Supporting Information
©Wiley-VCH 2024
69451 Weinheim, Germany

Saarvienin A – A Novel Glycopeptide with Potent Activity against Drug-resistant Bacteria

Amninder Kaur,^[a],[b],+^ Jaime Felipe Guerrero-Garzón,^[c],+^ Sari Rasheed,^[a],[b],+^ Martin Zehl,^[d]^ Franziska Fries,^[a],[b]^ Bernd Morgenstern,^[e]^ Sergey B. Zotchev,^[c]^* and Rolf Müller^[a],[b],[f]^*

**Abstract:** A member of a new family of glycopeptides, named saarvienin A, was isolated from a rare actinomycete *Amycolatopsis* sp. YIM10. Extensive NMR and MS analyses revealed a halogenated peptide core comprising four amino acids cyclized via a ureido linkage with an exocyclic 2-hydroxy-3-(4-hydroxyphenyl)propyl residue connected to a five-sugar/aminosugar chain. Two of the three aminosugars constitute the *N*-methylated and *N*,*O*-dimethylated derivatives of eremosamine (4-*epi*-vancosamine) that have not been reported in any natural product. Saarvienin A exhibits potent activity against a range of Gram-positive bacteria, effectively overcoming resistance to several frontline antibiotics in clinical isolates. It demonstrates an eight-fold reduction in minimum inhibitory concentrations (MICs) against methicillin-resistant, vancomycin-intermediate, and daptomycin-resistant *Staphylococcus aureus* compared to vancomycin.

DOI:

Table of Contents

[Table of Contents 2](#_Toc186446364)

[List of Figures 2](#_Toc186446365)

[List of Tables 3](#_Toc186446366)

[S1. General Information and Materials 4](#_Toc186446367)

[S2. Experimental Procedures 5](#_Toc186446368)

[Preliminary identification of Saarvienin A 5](#_Toc186446369)

[Growth and fermentation conditions of *Amycolatopsis* sp. YIM10 5](#_Toc186446370)

[Extraction of Saarvienin A from the fermentation broth 5](#_Toc186446371)

[Isolation and Purification of Saarvienin A 5](#_Toc186446372)

[X-Ray Crystallography analysis of (2*S*,3*R*)-*β*-Me-Cl-Trp 5](#_Toc186446373)

[S3. Chemical Derivatization Methods 6](#_Toc186446374)

[Amino Acid Analysis using Advanced Marfey’s Method 6](#_Toc186446375)

[Sugar/Aminosugar analysis 6](#_Toc186446376)

[S4. Biological evaluation of Saarvienin A 7](#_Toc186446377)

[Antimicrobial susceptibility testing 7](#_Toc186446378)

[Cytotoxicity evaluation 7](#_Toc186446379)

[S5. List of Figures — NMR, LCMS, and XRD data 8](#_Toc186446380)

[S6. List of Tables — NMR, LCMS, bioactivity, and XRD data 30](#_Toc186446381)

[References 47](#_Toc186446382)

[Author Contributions 47](#_Toc186446383)

List of Figures

[**Figure S1.** LC-MS analysis of the methanol extract from the freeze-dried culture of *Amycolatopsis* sp. YIM10 fermented in 5288 medium. Saarvienin A is produced at higher titers under these conditions as shown by the comparison of the positive ion mode base peak chromatogram (A) and the extracted ion chromatogram (B) of its [M+3H]^3+^ ion (*m/z* 503.9104±0.0050). 8](#_Toc192859164)

[**Figure S2.** High resolution ESI-Qq-TOF mass spectrum of saarvienin A (**A**) in comparison to the simulated isotopic pattern of the [M+2H]^2+^ ion of a compound with the sum formula C_73_H_105_ClN_10_O_22_ (**B**). 9](#_Toc192859165)

[**Figure S3.** High resolution ESI-Qq-TOF MS/MS spectrum of the [M+2H]^2+^ ion of saarvienin A (**UniVie**). 9](#_Toc192859166)

[**Figure S4.** ^1^H NMR spectrum of saarvienin A (**1**; 4-5 mg) in CD_3_OD/D_2_O (3:1) mixture (500 MHz). DMSO peak is observed in addition to solvent signals. 10](#_Toc192859167)

[**Figure S5.** ^13^C NMR spectrum of saarvienin A (**1**; 4-5 mg) in CD_3_OD/D_2_O mixture (125 MHz). Formic acid and DMSO peaks are observed in addition to solvent signals. 10](#_Toc192859168)

[**Figure S6.** ^1^H NMR spectrum of saarvienin A (**1**; 2 mg) in CD_3_OD/D_2_O (3:1) mixture (700 MHz). DMSO peak is observed in addition to solvent signals. 11](#_Toc192859169)

[**Figure S7.** ^1^H NMR spectrum of saarvienin A (**1**; 1.2 mg) in CD_3_OD/D_2_O (3:1) mixture after the addition of 10 µL formic acid-*d*_2_ (700 MHz). 11](#_Toc192859170)

[**Figure S8.** COSY NMR spectrum of saarvienin A (**1**; 4-5 mg) in CD_3_OD/D_2_O (3:1) mixture (500 MHz). 12](#_Toc192859171)

[**Figure S9.** TOCSY NMR spectrum of saarvienin A (**1**; 4-5 mg) in CD_3_OD/D_2_O (3:1) mixture (500 MHz). 13](#_Toc192859172)

[**Figure S10.** HSQC NMR spectrum of saarvienin A (**1**; 4-5 mg) in CD_3_OD/D_2_O (3:1) mixture (500 MHz). 14](#_Toc192859173)

[**Figure S11.** HMBC NMR spectrum (CNST = 6 Hz) of saarvienin A (**1**; 4-5 mg) in CD_3_OD/D_2_O (3:1) mixture (500 MHz). 15](#_Toc192859174)

[**Figure S12.** ROESY NMR spectrum of saarvienin A (**1**; 4-5 mg) in CD_3_OD/D_2_O (3:1) mixture (500 MHz). 16](#_Toc192859175)

[**Figure S13.** HMBC (CNST = 4 (a), 6 (b), 10 (c), and 12 Hz (d)) correlations to the carbonyl carbons of saarvienin A (**1**; 4-5 mg) in CD_3_OD/D_2_O (3:1) mixture (500 MHz). 17](#_Toc192859176)

[**Figure S14.** HMBC (CNST = 4 (a) and 6 (b) Hz) correlations to the carbonyl carbons of saarvienin A (**1**; 2.1 mg) in CD_3_OD/D_2_O (3:1) mixture (700 MHz). As the carbon NMR spectrum was acquired at 125 MHz using 4-5 mg sample, signal alignment may not be perfect. 18](#_Toc192859177)

[**Figure S15.** ^1^H NMR spectrum of saarvienin A (**1**; 0.8 mg) in DMSO-*d*_6_ (700 MHz). 18](#_Toc192859178)

[**Figure S16.** Key spin systems established using TOCSY NMR data of saarvienin A in DMSO-*d*_6_ (700 MHz). 19](#_Toc192859179)

[**Figure S17.** TOCSY NMR data at two different D9 values (0.09 and 0.03) to determine the alpha or beta characteristic of the NH protons of DAPA units in saarvienin A in DMSO-*d*_6_ (700 MHz). 20](#_Toc192859180)

[**Figure S18.** NOESY correlations between beta-NH protons of the DAPA units of saarvienin A in DMSO-*d*_6_ (700 MHz). 21](#_Toc192859181)

[**Figure S19.** Key *J*_HH_ coupling constants and ROESY correlations used to determine the relative configuration of the sugars units of saarvienin A. Dashed arrows represent weak correlations. 22](#_Toc192859182)

[**Figure S20.** Crystal Structure of (2*S*,3*R*)-*β*-Me-Cl-Trp unit isolated as an individual component from one of the extract fractions. 22](#_Toc192859183)

[**Figure S21.** Base peak chromatogram (LCMS data) and HRMSMS data of purified saarvienin A (HIPS). 23](#_Toc192859184)

[**Figure S22.** Key HMBC (→)/ROESY (↔) correlations and HR-MS/MS data used to establish the connectivity of various units in saarvienin A. 24](#_Toc192859185)

[**Figure S23.** LC-MS/MS data for the fragments observed after partial acidic hydrolysis (6N HCl, 110 °C, 20 min) of Saarvienin A. 25](#_Toc192859186)

[**Figure S24.** Absolute configuration of amino acids determined using Marfey’s method via comparison of the retention times and MS data of the derivatized amino acids (DAPA, HHPP, and Phe) in the hydrolysate (hydrolysis using 6N HCl; 110 °C; 24 h) to those of the derivatized standard amino acids. Extracted-ion chromatograms (LCMS) are shown. Trp degradation and DAPA racemization was observed upon hydrolysis under these conditions. 26](#_Toc192859187)

[**Figure S25.** Absolute configuration of (2*S*,3*R*)-*β*-Me-Cl-Trp determined using Marfey’s method. Extracted-ion chromatograms (LCMS) are shown. Peaks for racemized tryptophan derivative are observed at 21.5 min (with l-FDLA) and 19.2 min (with d-FLDA), as expected upon hydrolysis (24 h; 110 °C) using 5N NaOH solution. 26](#_Toc192859188)

[**Figure S26.** Absolute configuration of DAPA units determined using Marfey’s method after hydrolysis in DCl/D_2_O (20%) mixture (18 h; 110 °C). Green (disubstituted derivatives) and orange (monosubstituted derivatives) peaks represent extracted-ion chromatograms (LCMS) of the different monoisotopic ions of the FDLA derivatives of DAPA, while red (disubstituted derivatives) and blue chromatograms (monosubstituted derivatives) are the corresponding 1^st^ isotope ions. Presence of only the 1^st^ isotope ion for d-DAPA and a mixture of monoisotopic and 1^st^ isotope ions for l-DAPA residues suggests that d-DAPA is formed from l-DAPA via racemization upon hydrolysis in 6 N HCl. 27](#_Toc192859189)

[**Figure S27.** Absolute configuration of eremosamine (4-*epi*-vancosamine) determined via derivatization using *S/R*-2-butanol after hydrolysis in 1 N HCL (4 h; 60 °C). Extracted-ion chromatograms (LCMS) are shown. 28](#_Toc192859190)

[**Figure S28.** Absolute configuration of rhamnose determined via derivatization using *S/R*-2-butanol after hydrolysis in 1 N HCL (4 h; 60 °C). Extracted-ion chromatograms (LCMS) are shown. The assignments were also further confirmed by GCMS analysis after silylation of the butyl derivatives. 28](#_Toc192859191)

[**Figure S29.** Absolute configuration of digitoxose determined via derivatization using *S/R*-α-methoxy-α-(trifluoromethyl)phenylacetyl chloride (MTPACl) after hydrolysis in 1 N HCL (4 h; 60 °C). Extracted-ion chromatograms (LCMS) are shown. 29](#_Toc192859192)

[**Figure S30.** Absolute configuration of rhamnose determined via derivatization using *S/R*-α-Methoxy-α-(trifluoromethyl)phenylacetyl chloride (MTPACl) after hydrolysis in 1 N HCL (4 h; 60 °C). Extracted-ion chromatograms (LCMS) are shown. 29](#_Toc192859193)

List of Tables

[**Table S1**. 1D and 2D NMR data of saarvienin A (**1**; 4-5 mg) in CD_3_OD/D_2_O (3:1) mixture (500 MHz). 30](#_Toc192423574)

[**Table S2.** Absolute configuration of amino acids determined using Marfey’s method via comparison of the retention times (min) and MS data of the derivatized amino acids in the hydrolysate to those of the derivatized standard amino acids. 33](#_Toc192423575)

[**Table S3.** Absolute configuration of sugars determined via comparison of the retention times (min) and MS data of the *R*/*S*-2-butyl derivatives of sugars in the hydrolysate to those of standard sugars. 33](#_Toc192423576)

[**Table S4.** Absolute configuration of sugars determined via comparison of the retention times (min) and MS data of the *R*/*S*-α-methoxy-α-(trifluoromethyl)phenylacetyl (MTPA) derivatives of sugars in the hydrolysate to those of standard sugars. 33](#_Toc192423577)

[**Table S5**. Minimum inhibitory concentrations (MICs) of saarvienin A against a collection of vancomycin-resistant *E. faecium* clinical isolates with reduced susceptibility to daptomycin [n=26]. Values represent the mean of two independent experiments. 34](#_Toc192423578)

[**Table S6.** Genes in the predicted saarvienin A biosynthesis gene cluster and their putative products and functions. 35](#_Toc192423579)

[**Table S7**. Crystal data and structure refinement for (2*S*,3*R*)-*β*-Me-Cl-Trp. 36](#_Toc192423580)

[**Table S8**. Atomic coordinates (x 104) and equivalent isotropic displacement parameters (Å2x 103) for (2*S*,3*R*)-*β*-Me-Cl-Trp. U(eq) is defined as one third of the trace of the orthogonalized Uij tensor. 37](#_Toc192423581)

[**Table S9**. Bond lengths [Å] and angles [°] for (2*S*,3*R*)-*β*-Me-Cl-Trp. 39](#_Toc192423582)

[**Table S10**. Anisotropic displacement parameters (Å2x 103) for (2*S*,3*R*)-*β*-Me-Cl-Trp. The anisotropic displacement factor exponent takes the form: -2π2[h2 a*2U11 + ... + 2 h k a* b* U12] 41](#_Toc192423583)

[**Table S11**. Hydrogen coordinates (x 104) and isotropic displacement parameters (Å2x 10 3) for (2*S*,3*R*)-*β*-Me-Cl-Trp. 43](#_Toc192423584)

[**Table S12**. Torsion angles [°] for (2*S*,3*R*)-*β*-Me-Cl-Trp. 45](#_Toc192423585)

[**Table S13**. Hydrogen bonds for (2*S*,3*R*)-*β*-Me-Cl-Trp [Å and °]. 46](#_Toc192423586)

S1. General Information and Materials

**Instrumentation.** Aqueous mobile-phases for semi-preparative HPLC were prepared using water purified with a Milli-Q^®^ Integral water purification system (purified to a resistivity of 18.2 MΩ.cm at 25°C). HPLC-grade acetonitrile (ACN), methanol (MeOH), and formic acid (FA) were used. Mobile-phases for analytical HPLC were prepared using water distilled over a vertical double distillation apparatus, LC-MS grade organic solvents, and LC-MS grade formic acid.

**NMR** spectra were recorded on a Bruker Ascend 700 (^1^H at 700 MHz, ^13^C at 175 MHz) and Bruker Ultra Shield 500 (^1^H NMR data at 500 MHz, ^13^C at 125 MHz) spectrometers (Bruker Corporation, Billerica, Massachusetts, USA) equipped with 5-mm TCI cryoprobe heads using standard pulse programs. Chemical shifts are expressed in parts per million (ppm) in CD_3_OD/D_2_O (~3:1) mixture (*δ*_H_ = 3.31, *δ*_C_ = 49.15) and DMSO (*δ*_H_ = 2.50, *δ*_C_ = 39.51). Multiplicities are described as s (singlet), d (doublet), q (quadruplet), dd (doublet of doublets), ddd (doublet of doublets of doublets), t (triplet), dd (doublet of triplets), m (multiplet), and br (broad). Coupling constants *J* are reported in Hertz (Hz) to the nearest 0.1 Hz.

**LC-MS analyses at the University of Vienna (UniVie)** were performed on a Vanquish Horizon UHPLC system (Thermo Fisher Scientific) coupled to the ESI source of a timsTOF fleX mass spectrometer (Bruker Daltonics) using an Acquity Premier HSS T3 column, 2.1 x 150 mm, 1.8 µm (Waters). The detailed conditions and parameters were reported previously.^[1]^ **Analytical LC-MS analyses** **at HIPS, Saarbrücken** were performed using a Dionex Ultimate 3000 UPLC system (Thermo Fisher Scientific Inc., Waltham, Massachusetts, USA) using a Waters BEH C18 (100 x 2.1 mm, 1.7 µm), equipped with a DAD module, and coupled to a Bruker maXis 4G UHR-TOF mass spectrometer (Bruker Daltonics) with electrospray ionization (ESI) and MS^2^ capabilities (at HIPS, Saarbrücken). High-resolution mass spectra (HRMS) and MS^2^ fragmentation spectra were recorded using the same systems as for LC-MS. The following chromatographic systems were used --- **Method A1**: temperature of 45°C, flow rate of 0.6 mL/min with ACN/0.1% FA and H_2_O/0.1% FA [5% ACN (0.5 min), linear gradient from 5 to 95% of ACN (18 min), 95% ACN (2 min) followed by re-equilibration to the starting conditions], and UV detection in the range from 200 to 600 nm; **Method A2**: temperature of 45°C, flow rate of 0.6 mL/min with ACN/0.1% FA and H_2_O/0.1% FA [5% ACN (0.5 min), linear gradient from 5 to 95% of ACN (9 min), 95% ACN (2 min) followed by re-equilibration to the starting conditions], and UV detection in the range from 200 to 600 nm. LCMS and MS^2^ fragmentation data were visualised and analysed using Bruker DataAnalysis software. **Marfey’s LCMS analysis** (**Method B**) employed an Acquity BEH C18 (2.1 x 100 mm, 1.7 µm) column with a flow rate of 0.55 mL/min with the following gradient --- Solvent A: H_2_O + 0.1% FA; solvent B: ACN + 0.1% FA; gradient: 0-1 min: 5-10% B, 1-15 min: 10-35% B, 15-22 min: 35-50% B, 22-25 min: 50-80% B. Detection was achieved by DAD and UHPLC maXis4G qTOF HRMS in positive mode. **Sugar/amino sugar LCMS analysis** (**Method C**) was also performed using an Acquity BEH C18 (2.1 x 100 mm, 1.7 µm) column with a flow rate of 0.6 mL/min with the following gradient --- Solvent A: H_2_O + 0.1% FA; solvent B: ACN + 0.1% FA; gradient: 0-1 min: 5-10% B, 1-22 min: 10% B, 22-25 min: 10-80% B, 25-26 min: 80% B, 26-26.5 min: 80-5% B, 26.5-31 min: 5% B. **Mosher derivatives of digitoxose and rhamnose** were separated using **LCMS Method D** employing an Acquity BEH C18 (2.1 x 100 mm, 1.7 µm) column with a flow rate of 0.6 mL/min with the following gradient --- Solvent A: H_2_O + 0.1% FA; solvent B: ACN + 0.1% FA; gradient: 0-5 min: 5-65% B, 5-20.5 min: 65% B, 20.5-21 min: 65-95% B, 21-26 min: 95% B, 26-26.5 min: 95-5% B, 26.5-31 min: 5% B. **Flash chromatography** was performed using Biotage Isolera One system employing a Biotage® Sfär C18 D Duo 100 Å 30 µm 60 g column.The conditions for chromatographic system **FC1** used were as follows: solvent A: H_2_O + 0.1% FA, solvent B: MeOH + 0.1% FA; gradient: 5 column volumes (CV): 5% B, 30 CV: 5-95% B, 5 CV: 95-100% B, 10 CV: 100% B. Every 3 tubes were pooled and analyzed using LCMS to identify the fractions containing saarvienin A.

**Preparative RP-HPLC** was performed using a Waters Autopurifier system (Waters Corporation, Milford, Massachusetts, USA) equipped with a DAD detector module and a single-quad MS spectrometer using a waters XBridge Prep C18 column (10μm, 19 x 150 mm) at 25 mL/min flow rate, and fractions were collected automatically by time-based collection and their purity was verified by analytical LC-MS. The conditions for chromatographic system **P1** used were as follows: solvent A: H_2_O + 0.1% FA, solvent B: MeOH + 0.1% FA; gradient: 0-6 min: 5-45% B, 6-25 min: 45-55% B, 25-26 min: 55-95% B, 25-27.5 min: 95% B, 27.5-28.5 min: 95-55% B, 28.5-30 min: 5% B.

**Semi-preparative RP-HPLC** was performed using a Dionex Ultimate system (Thermo Fisher) equipped with a DAD detector, and XSelect CSH 130 Prep C18 column (10um; 10 x 250 mm) at 40°C and 5 mL/min flow rate. Compounds were collected automatically by time-based collection and their purity was verified by analytical LC-MS. The conditions for chromatographic system **SP1** used were as follows: solvent A: H_2_O + 0.1% FA, solvent B: MeOH + 0.1% FA; gradient: 0-5 min: 5-50% B, 5-11 min: 50% B, 11-11.5 min: 50-95% B, 11.5-15.5 min: 95% B, 15.5-16 min: 95-5% B, 16-20 min: 5% B. UV detection at 190, 220, 254, and 280 nm. The conditions for chromatographic system **SP2** used were as follows: solvent A: H_2_O + 0.1% FA, solvent B: MeOH + 0.1% FA; gradient: 0-11 min: 35% B, 11-11.5 min: 50-95% B, 11.5-15.5 min: 95% B, 15.5-16 min: 95-35% B, 16-20 min: 35% B. UV detection at 190, 220, 254, and 280 nm.

**GC-MS** analysis was done using a 6890N gas chromatograph (Agilent) equipped with a 5973 mass detector, a 7683B injector, a HP-1 ms column (Agilent, 100% dimethylpolysiloxane, 0.1 μm film, 30 m length, 0.25 mm ID), and helium as the carrier gas. General settings were as follows: inlet temperature of 275 °C; GC-MS transfer line of 280 °C, ion source of 230 °C, and quadrupole of 150 °C; the MS detector was operated in the scan mode (40–700 *m*/*z*). GC was performed with 1 mL/min carrier gas flow, and 1 μL of the sample was injected in the split mode (1:10). The column oven temperature protocol was as follows: held at 50 °C for 2.5 min, increased from 50 to 180 °C with a 5 °C/min rate, held at 180 °C for 10 min, and decreased to 50 °C with a rate of 30 °C/min, which was held for 5 min.

S2. Experimental Procedures

Preliminary identification of Saarvienin A

Saarvienin A was initially detected in the course of a previous study on secondary metabolites of *Amycolatopsis* sp. YIM10.^[2]^ It was found as minor impurity in a fraction containing mainly 1,2,4-trimethoxynaphthalene and characterized as novel chlorinated glycopeptide by LC-MS. Saarvienin A was detected with moderate abundance in small scale liquid and solid media cultures, but the upscaling of the fermentation and development of a purification protocol turned out to be challenging.

One attempt to facilitate chemoselective purification by affinity chromatography was based on the hypothesis that saarvienin A is related to vancomycin and could bind to the d-alanyl-d-alanine peptide motif found in bacterial peptidoglycan precursors. To that end, 15 mg d-Ala-d-Ala ligand were coupled to 2 mL of NHS-activated Sepharose 4 Fast Flow (Cytiva^®^) for preparation of affinity columns as described by the Cytiva^®^ protocol. *Amycolatopsis* sp. YIM10 fermentation broth and vancomycin (2 mg/mL in 0.02M phosphate buffer, pH 7.0), used as control, were treated and extracted using a respective Sepharose 4 Fast Flow-d-Ala-d-Ala affinity column (2 mL).^[3]^ The obtained fractions were freeze-dried and dissolved in methanol for further HPLC and LC-MS analysis as well as antimicrobial activity testing. However, in contrast to vancomycin, saarvienin A did not show affinity for the Sepharose-bound d-Ala-d-Ala.

Finally, after testing different methods and careful optimisation of the conditions, the following protocol was used to obtain sufficient amounts of saarvienin A for the full structure elucidation and biological evaluation.

Growth and fermentation conditions of *Amycolatopsis* sp. YIM10

*Amycolatopsis* sp. YIM10 was grown in HA agar (10 g malt extract, 4 g yeast extract, 4 g glucose, 1.46 g CaCl_2_, 16 g agar, tap water up to 1 L, pH adjusted to 7.3) at 28 °C during 14 days to harvest spores. 10 mL of *Amycolatopsis* seeding medium (ASM) (15 g glucose, 15 g glycerol, 15 g soya peptone, 3 g NaCl, 5 g yeast extract, in 1 L of distilled water) were inoculated with 100 µL of dense spore suspension and cultivated in a 100 mL flask at 30 °C with 200 rpm for 4 days. 250 mL baffled flasks containing 50 mL ASM were inoculated with 3 mL of dense YIM10 seeding culture and cultivated at 30 °C with 200 rpm for 4 days. 250 mL baffled flasks containing 50 mL 5288^[2]^ were inoculated with 2 mL of well-grown YIM10 seeding culture and cultivated at 30 °C with 200 rpm for 14 days.

Extraction of Saarvienin A from the fermentation broth

The *Amycolatopsis* sp. YIM10 fermentation culture was freeze-dried. 600 mL methanol/L of fermentation broth were used to extract the dried material. Extraction was carried out at room temperature with 200 rpm for 1 hour. The methanolic extract was filtered using 180 µm pore size paper filter and the methanol was evaporated under reduced pressure to generate the *Amycolatopsis* sp. YIM10 crude extract. Antimicrobial activity was monitored using the disk diffusion assay with *Staphylococcus carnosus* DZMZ 20501 and *Bacillus subtilis* 168 as test organisms. LC-MS (**UniVie**) was used to monitor the production of saarvienin A, which was detected as one of the most abundant peaks under these optimised growth conditions (Figures S1-S3).

Isolation and Purification of Saarvienin A

The crude extract (~20 g) obtained from UniVie was first subjected to liquid-liquid partitioning between water and dichloromethane (3 × 200 ml; DCM fraction = 850 mg). The glycopeptide was extracted into the water layer, which was further extracted from the latter using butanol (3 × 200 ml). The butanol fraction (2 g) was dried and subjected to reversed-phase (RP) flash column chromatography (method **FC1**) or preparative (method **P1**) HPLC (see section S1 for method details). For flash column chromatography fractions, every 3 tubes were pooled and analyzed using LCMS (method **A2**) to identify the fractions containing saarvienin A. Enriched fractions containing saarvienin A were thereafter processed via semi-prep RP HPLC using method **SP1** to yield ~22 mg of pure saarvienin A (*t*_R_ = 9-10 min). Similarly, fractions containing (2*S*,3*R*)-*β*-Me-Cl-Trp were subjected to semi-prep RP HPLC using method **SP2** to yield ~10 mg of the pure compound (*t*_R_ = 8-9 min), which was characterized using NMR, XRD, and optical rotation measurements and used as a standard to determine the absolute configuration of *β*-Me-Cl-Trp unit present in saarvienin A.

X-Ray Crystallography analysis of (2*S*,3*R*)-*β*-Me-Cl-Trp

**Crystallization conditions:** Crystals of (2*S*,3*R*)-*β*-Me-Cl-Trp were first obtained in 0.1N HCl, but these showed heavy twinning characteristics. The same sample was dried, dissolved in methanol, and left standing in a glass vial with loose cap for 2-3 days to afford small crystals of this compound.

**Crystallographic data**: The data set was collected using a Rigaku XtaLAB Synergy-S diffractometer with a microfocus sealed tube and a HyPix-6000HE Hybrid Photon Counting (HPC) detector. Monochromated Mo_Kα_ radiation (λ = 0.71073 Å) was used. Data were collected at 130(2) K and corrected for absorption effects using the multi-scan method. The structure was solved by direct methods using SHELXT^[4]^ and was refined by full matrix least squares calculations on F^2^ (SHELXL2019^[5]^) in the graphical user interface Shelxle^[6]^.

**Refinement**: All non H-atoms were located in the electron density maps and refined anisotropically. C-bound H atoms were placed in positions of optimized geometry and treated as riding atoms. Their isotropic displacement parameters were coupled to the corresponding carrier atoms by a factor of 1.2 (CH) or 1.5 (CH3). The positional parameters of the O- and N-bonded H-atoms were refined using isotropic displacement parameters which were set at 1.2 and 1.5 times the Ueq value of the parent atoms, respectively. In addition, restraints of 0.84 (0.01) and 0.88 (0.01) Å were used for the O-H and N-H bond lengths, respectively. However, it must be mentioned that the quality of the scattering contribution of the crystal water molecules in the unit cell was somewhat weaker than for the rest of the structure.

**NMR Data (CD_3_OD/D_2_O; 500/125 MHz)**: ^1^H [*δ*_H_ (mult., *J*, #H)] 1.41 (d, 7.1, H_3_-13), 3.91 (dq, 3.4, 7,1, H-3), 3.97 (br d, 3.4, H-2), 7.11 (dd, 2.0, 8.6, H-9), 7.24 (s, H-5), 7.35 (d, 8.6, H-8), 7.83 (d, 2.0, H-11). ^13^C [*δ*_H_ (*J*, #H)] 13.7 (C-13), 32.9 (C-3), 60.1 (C-2), 113.9 (C-8), 115.8 (C-4), 119.2 (C-11), 123.2 (C-9), 125.7 (C-5), 126.1 (C-10), 128.8 (C-12), 137.1 (C-7), 173.6 (C-1). [α]_D_^23^ – 30.4 (*c* = 0.25, 0.1 M HCl).

S3. Chemical Derivatization Methods

Amino Acid Analysis using Advanced Marfey’s Method

To determine the absolute configuration of amino acids, advanced Marfey’s method was used.^[7]^ Approximately 0.1-0.2 mg of saarvienin A was hydrolyzed using 6 N HCl (0.2 mL) at 110 °C for 1, 6, or 18-24 h in glass vials. Samples were dried overnight under nitrogen and dissolved in H_2_O (110 μL). They were split into two 50 μL aliquots, and 1 N NaHCO_3_ (20 μL) and 1% 1-fluoro-2,4-dinitrophenyl-5-leucine-amide (l-FDLA or d-FDLA solution in acetone, 20 μL) were added individually. The mixtures were heated to 40 °C for 2 h and cooled down to RT, and the reaction was quenched by adding 2 N HCl (10 μL). After adding 300 µL of ACN/MeOH mixture, the samples were centrifuged and subsequently analyzed by LCMS (method **B**). Amino acid standards were derivatized in a similar manner. The retention times are included in Table S2.

**Note**: In some cases, very slow reaction of DAPA units in saarvienin A (after hydrolysis) with Marfey’s reagent was observed. Only minor peaks in LCMS data were observed upon analyzing immediately after the reaction. Prominent peaks were only detected after the reaction mixture was re-analysed by LCMS after a few days.

**Absolute Configuration of DAPA Units**: DAPA is known to racemize during acidic hydrolysis. To determine the presence of d- and/or l-DAPA units and provide evidence for the racemization of this amino acid, saarvienin A was hydrolyzed using DCl/D_2_O (20 wt% in D_2_O) at 110 °C for 6 and 18 h in glass vials. The sample was further subjected to Marfey’s analysis (LCMS method **B**). The procedure was similar to that described above.

**Absolute Configuration of *β*-Me-Cl-Trp**. As Trp is known to degrade under acidic conditions, basic hydrolysis of saarvienin A was done using 5 N NaOH solution (110 °C, 18 h) in glass vials. The reaction mixture was quenched/neutralized using HCl, and the hydrolysate was subjected to solid-phase extraction (SPE), where Trp via elution with 50% ACN through a C18 SPE cartridge. The sample was dried and further subjected to Marfey’s analysis (LCMS method **B**) using a similar procedure as described above. The retention times are included in Table S2.

Sugar/Aminosugar analysis

To determine the absolute configuration of sugars and amino sugars, 0.2-0.3 mg of saarvienin A was hydrolyzed using 1 N HCl (0.5 mL) at 60 °C for 4 h in a glass vial. Samples were dried overnight under nitrogen. The residue was separately dissolved in 0.2 mL (*S*)- or (*R*)-2-butanol and 5 μL acetyl chloride, and the reaction mixture was stirred at 80 °C for 8 h. The reaction mixture was centrifuged and directly analyzed using LCMS (LCMS method **C**). Authentic standard of l-rhamnose was derivatized with (*S*)- or (*R*)-2-butanol and analyzed by LCMS to compare the retention times (Table S3). For GCMS analysis, the reaction was quenched with 0.5 mL saturated aqueous NaHCO_3_, the solution was extracted two times with 0.4 mL ethyl acetate. After the ethyl acetate layer was dried, silylation was conducted by dissolving the residue in 40 μL 1-(trimethylsilyl)imidazole and 160 μL pyridine and stirring the solution at 60 °C for 15 min. The mixture was dried, and extracted two times with 0.4 mL ethyl acetate and water (1:1 *v*/*v*). The organic layer was dried over MgSO_4_ and analyzed by GC-MS. Although silylated rhamnose derivatives could be detected by GCMS, their yields were rather low. To obtain eremosamine, the natural product eremomycin was purchased from MedChemExpress (New Jersey, USA), and 0.2-0.3 mg of it was subjected to hydrolysis using 1 N HCl (0.5 mL) at 60 °C for 4 h in a glass vial. The hydrolysate was dried and subjected to butanolysis using (*S*)- or (*R*)-2-butanol under similar conditions as described above. Comparison of MS data and retention times (LCMS method **C**) allowed the identification of l-eremosamine and l-rhamnose. Unfortunately, butanol derivatives of digitoxose prepared in a similar manner could not be separated even after using several different HPLC columns. Their silylated derivatives could also not be located in GCMS data.

As the butanol derivatives of digitoxose could not be separated, we decided to use a derivatization agent with more bulky groups. Mosher’s reagent was readily available and the presence of fluoro and phenyl groups was expected to provide better resolution during LCMS analysis using Fluoro-Phenyl columns. However, these derivatives showed baseline resolution using normal C18 columns, not necessitating the use of Fluoro-Phenyl columns. For the analysis, 0.2-0.3 mg of saarvienin A was hydrolyzed using 1 N HCl (0.5 mL) at 60 °C for 4 h in a glass vial; note: hydrolysis using 6N HCl led to degradation of digitoxose. Samples were dried under nitrogen and the residue was dissolved in 0.15 mL pyridine-d_5_. To this solution, 20 µL of *S*-α-methoxy-α-(trifluoromethyl)phenylacetyl chloride (*S*-MTPACl) was added and reaction mixture was stirred overnight at 30 °C. The reaction mixture was centrifuged and directly analyzed using LCMS (LCMS method **C**). Authentic standards of d-digitoxose and l-rhamnose were derivatized with (*S*)- or (*R*)-MTPACl in a similar manner (using 100 µL pyridine-d_5_ and 10 µL of (*S*)- or (*R*)-MTPACl) and analyzed by **LCMS method D** to compare the retention times (Table S4).

S4. Biological evaluation of Saarvienin A

Antimicrobial susceptibility testing

All bacterial isolates were handled following standard procedures. *Staphylococcus aureus* strains Mu50 and N315 were obtained from the Institute of Medical Microbiology, Zurich, Switzerland*. Enterococcus faecalis* ATCC 29212, *S. aureus* ATCC 29213, *Mycobacterium tuberculosis* ATCC 25177and *Escherichia coli* ATCC 25922 were purchased from the American Type Culture Collection (ATCC), USA. *E. faecalis* DSM 12956 and *Enterococcus faecium* DSM 17050 were purchased from the German Collection of Microorganisms and Cell Cultures (DSMZ). The *S. aureus* HG001 strain and its daptomycin-resistant mutant, HG001 DAPR, were provided by the Institute for Pharmaceutical Microbiology (IPM), University of Bonn. *E. coli* WO153 was generously provided by the Antimicrobial Discovery Center, Department of Biology, Northeastern University. Additional strains were part of our internal collection and were cultured under conditions recommended by their respective depositors. A collection of twenty six vancomycin-resistant *E. faecium* clinical isolates with reduced sensitivity to daptomycin, were generously provided by PD Dr. Stefano Mancini from the Institute of Medical Microbiology, University of Zurich.

The minimal inhibitory concentrations (MICs) of Saarvienin A and vancomycin were determined using the standard broth microdilution method in Mueller-Hinton Broth II (cation-adjusted; Sigma). Briefly, 75 µL of bacterial suspension (~4 × 10⁵ colony-forming units (CFU)/mL) in Mueller-Hinton Broth II was added to each well of a 96-well plate, along with 75 µL of serially diluted compound solutions (starting from 64 µg/mL). The plates were incubated at 37 °C for 24 hours, and the MIC was reported as the lowest concentration that inhibited visible bacterial growth. For *M. tuberculosis* and *M. smegmatis*, MIC determination was performed in 7H9 complete medium (BD Difco; Becton Dickinson) supplemented with 10% oleic acid-albumin-dextrose-catalase (OADC; BD), as described previously.^[8]^ A single-cell suspension of *M. tuberculosis* in PBS was prepared to achieve a final concentration of 10⁵ CFU/mL. Saarvienin A and vancomycin were tested in two-fold serial dilutions in a final concentration of 1% DMSO. The MIC was visually assessed as the lowest concentration without visible growth. MIC of daptomycin was assessed in Mueller-Hinton-Broth II (cation adjusted) supplemented to 50 µg/mL Ca^2+^. *E. faecalis* ATCC29212 was used as reference strain.

Cytotoxicity evaluation

HepG2 cells (human hepatoblastoma cell line; ACC 180, DSMZ) were cultured according to the conditions recommended by the supplier, in Dulbecco's modified Eagle medium (DMEM) supplemented with 10% fetal bovine serum. To evaluate the antiproliferative activity of Saarvienin A, cells were seeded at 6 × 10³ cells per well in 96-well plates with 120 µL of complete medium. After a 2-hour equilibration period, serially diluted compounds were added in 60 µL of complete medium. The compounds, solvent control, and doxorubicin (used as a reference) were tested in duplicate in two independent experiments. Following 5 days of incubation, 20 µL of 5 mg/mL MTT (thiazolyl blue tetrazolium bromide) in PBS was added to each well, and the cells were further incubated at 37°C for 2 hours. The medium was then removed, and cells were washed with 100 µL PBS. To dissolve the formazan crystals, 100 µL of 2-propanol/10 N HCl (250:1) was added. Absorbance was measured at 570 nm using a microplate reader (Tecan Infinite M200Pro), and cell viability was calculated as a percentage relative to the solvent control. IC50 values were determined by sigmoidal curve fitting using GraphPad PRISM 8 (GraphPad Software).

S5. List of Figures — NMR, LCMS, and XRD data

**Figure S1.** LC-MS analysis of the methanol extract from the freeze-dried culture of *Amycolatopsis* sp. YIM10 fermented in 5288 medium. Saarvienin A is produced at higher titers under these conditions as shown by the comparison of the positive ion mode base peak chromatogram (A) and the extracted ion chromatogram (B) of its [M+3H]^3+^ ion (*m/z* 503.9104±0.0050).

**Figure S2.** High resolution ESI-Qq-TOF mass spectrum of saarvienin A (**A**) in comparison to the simulated isotopic pattern of the [M+2H]^2+^ ion of a compound with the sum formula C_73_H_105_ClN_10_O_22_ (**B**).

**Figure S3.** High resolution ESI-Qq-TOF MS/MS spectrum of the [M+2H]^2+^ ion of saarvienin A (**UniVie**).

**DMSO**

**Figure S4.** ^1^H NMR spectrum of saarvienin A (**1**; 4-5 mg) in CD_3_OD/D_2_O (3:1) mixture (500 MHz). DMSO peak is observed in addition to solvent signals.

**Figure S5.** ^13^C NMR spectrum of saarvienin A (**1**; 4-5 mg) in CD_3_OD/D_2_O mixture (125 MHz). Formic acid and DMSO peaks are observed in addition to solvent signals.

**Figure S6.** ^1^H NMR spectrum of saarvienin A (**1**; 2 mg) in CD_3_OD/D_2_O (3:1) mixture (700 MHz). DMSO peak is observed in addition to solvent signals.

**Figure S7.** ^1^H NMR spectrum of saarvienin A (**1**; 1.2 mg) in CD_3_OD/D_2_O (3:1) mixture after the addition of 10 µL formic acid-*d*_2_ (700 MHz).

**Figure S8.** COSY NMR spectrum of saarvienin A (**1**; 4-5 mg) in CD_3_OD/D_2_O (3:1) mixture (500 MHz).

**Figure S9.** TOCSY NMR spectrum of saarvienin A (**1**; 4-5 mg) in CD_3_OD/D_2_O (3:1) mixture (500 MHz).

**Figure S10.** HSQC NMR spectrum of saarvienin A (**1**; 4-5 mg) in CD_3_OD/D_2_O (3:1) mixture (500 MHz).

**Figure S11.** HMBC NMR spectrum (CNST = 6 Hz) of saarvienin A (**1**; 4-5 mg) in CD_3_OD/D_2_O (3:1) mixture (500 MHz).

**Figure S12.** ROESY NMR spectrum of saarvienin A (**1**; 4-5 mg) in CD_3_OD/D_2_O (3:1) mixture (500 MHz).

**b**

**a**

**d**

**c**

**Figure S13.** HMBC (CNST = 4 (a), 6 (b), 10 (c), and 12 Hz (d)) correlations to the carbonyl carbons of saarvienin A (**1**; 4-5 mg) in CD_3_OD/D_2_O (3:1) mixture (500 MHz).

**a**

**b**

**Figure S14.** HMBC (CNST = 4 (a) and 6 (b) Hz) correlations to the carbonyl carbons of saarvienin A (**1**; 2.1 mg) in CD_3_OD/D_2_O (3:1) mixture (700 MHz). As the carbon NMR spectrum was acquired at 125 MHz using 4-5 mg sample, signal alignment may not be perfect.

**Figure S15.** ^1^H NMR spectrum of saarvienin A (**1**; 0.8 mg) in DMSO-*d*_6_ (700 MHz).

**DAPA**

**Phe**

**HHPP**

**Trp**

**Figure S16.** Key spin systems established using TOCSY NMR data of saarvienin A in DMSO-*d*_6_ (700 MHz).


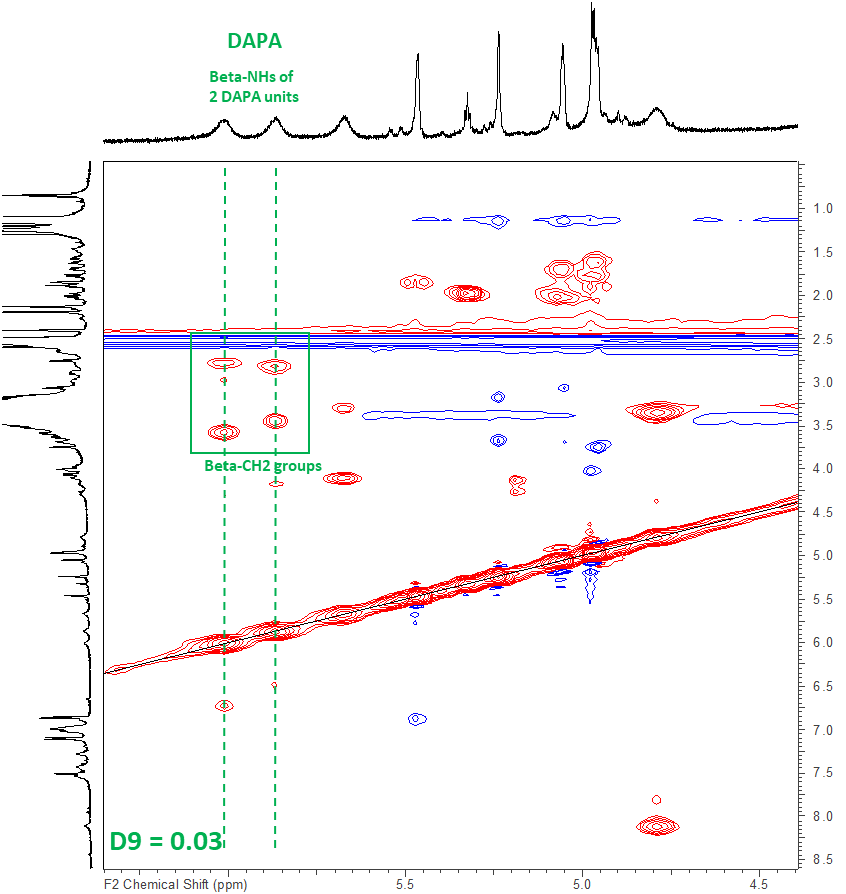

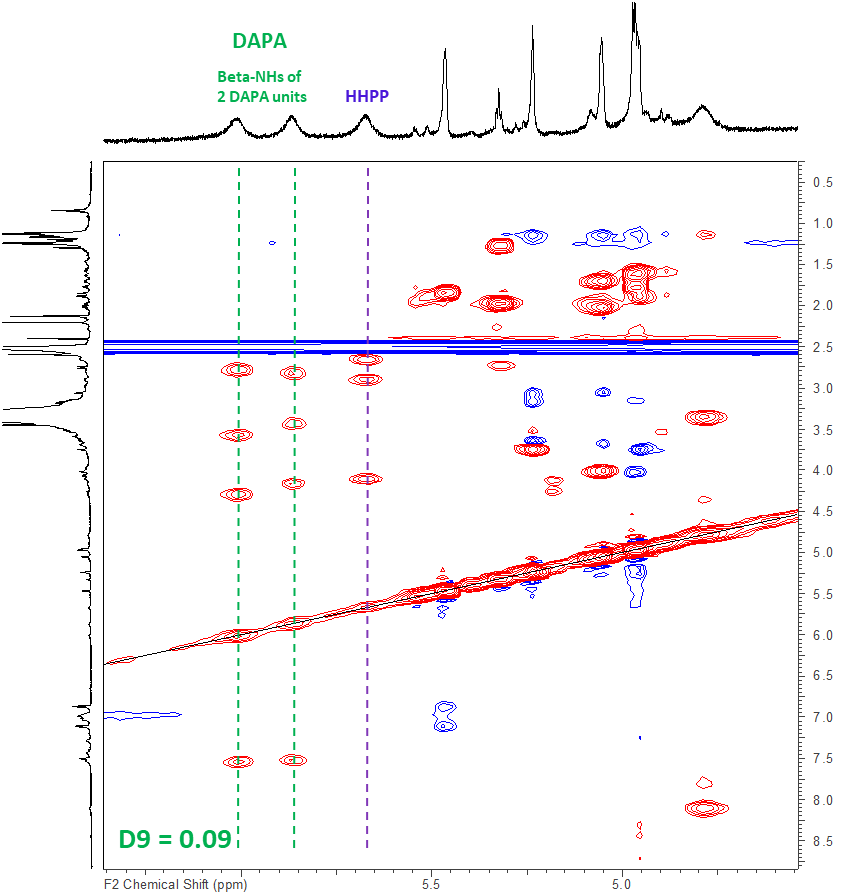


**Figure S17.** TOCSY NMR data at two different D9 values (0.09 and 0.03) to determine the alpha or beta characteristic of the NH protons of DAPA units in saarvienin A in DMSO-*d*_6_ (700 MHz).

**Figure S18.** NOESY correlations between beta-NH protons of the DAPA units of saarvienin A in DMSO-*d*_6_ (700 MHz).

**Figure S19.** Key *J*_HH_ coupling constants and ROESY correlations used to determine the relative configuration of the sugars units of saarvienin A. Dashed arrows represent weak correlations.


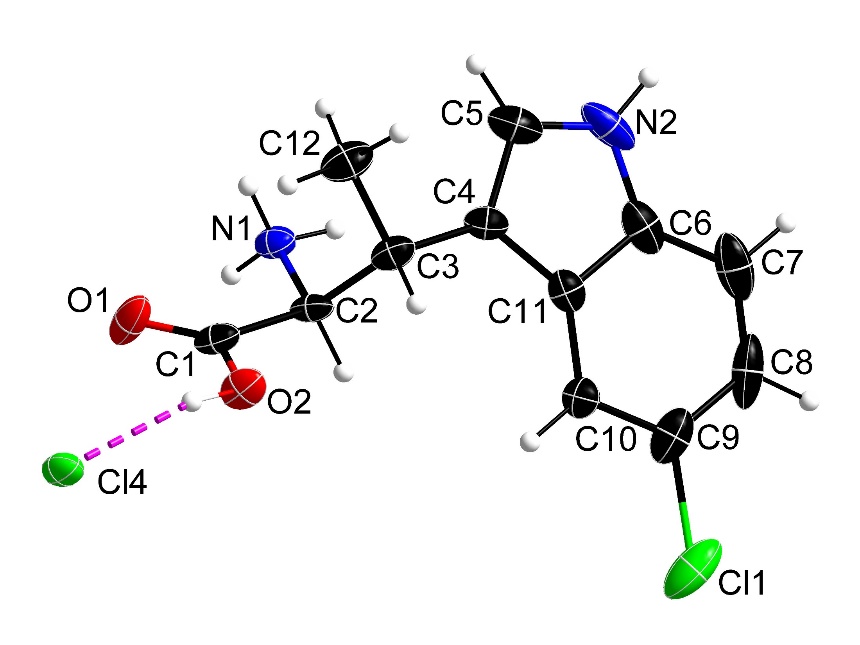


**Figure S20.** Crystal Structure of (2*S*,3*R*)-*β*-Me-Cl-Trp unit isolated as an individual component from one of the extract fractions.

Saarvienin A

290.1603

302.1965

**Figure S21.** Base peak chromatogram (LCMS data) and HRMSMS data of purified saarvienin A (HIPS).

**Figure S22.** Key HMBC (→)/ROESY (↔) correlations and HR-MS/MS data used to establish the connectivity of various units in saarvienin A.


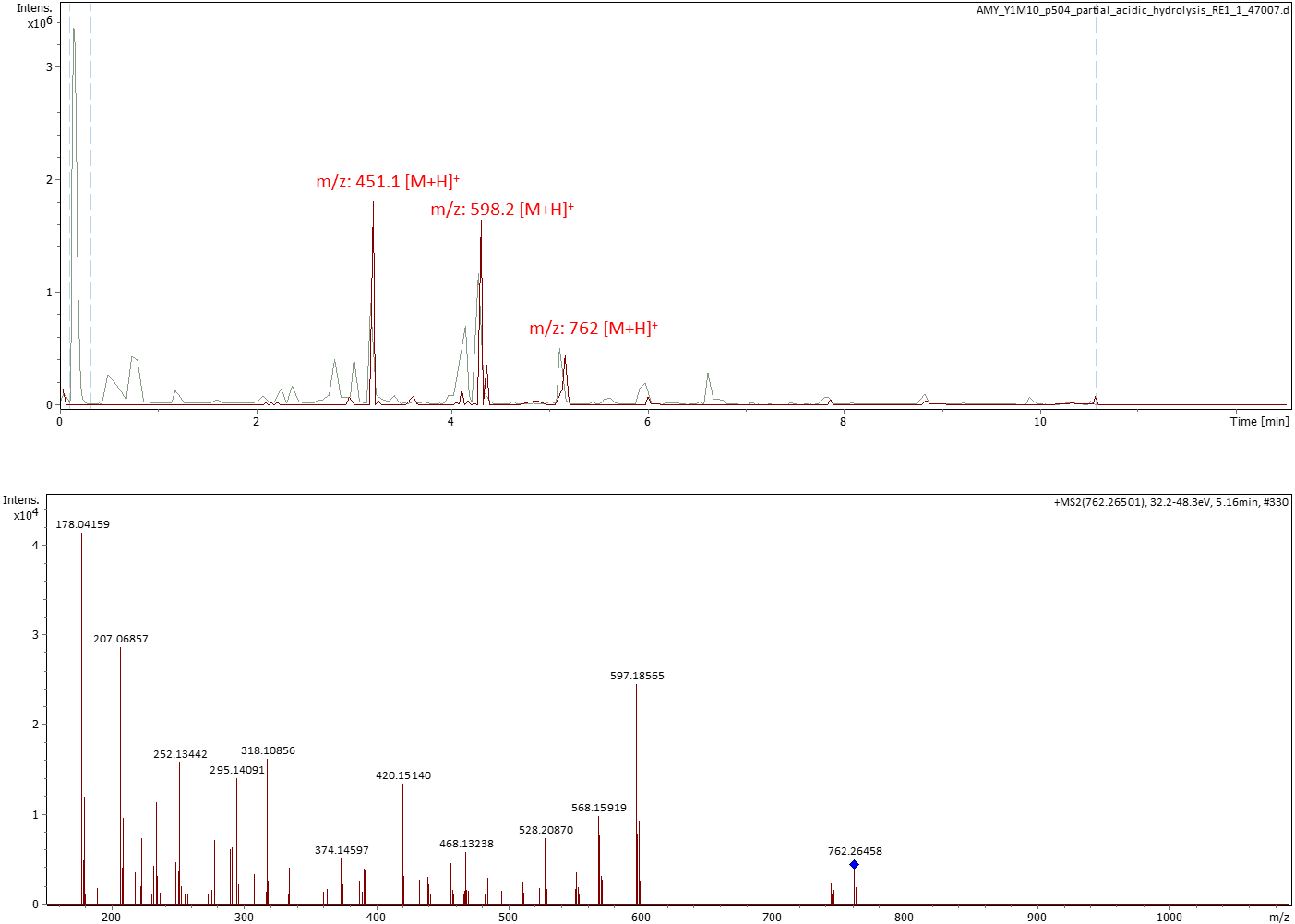

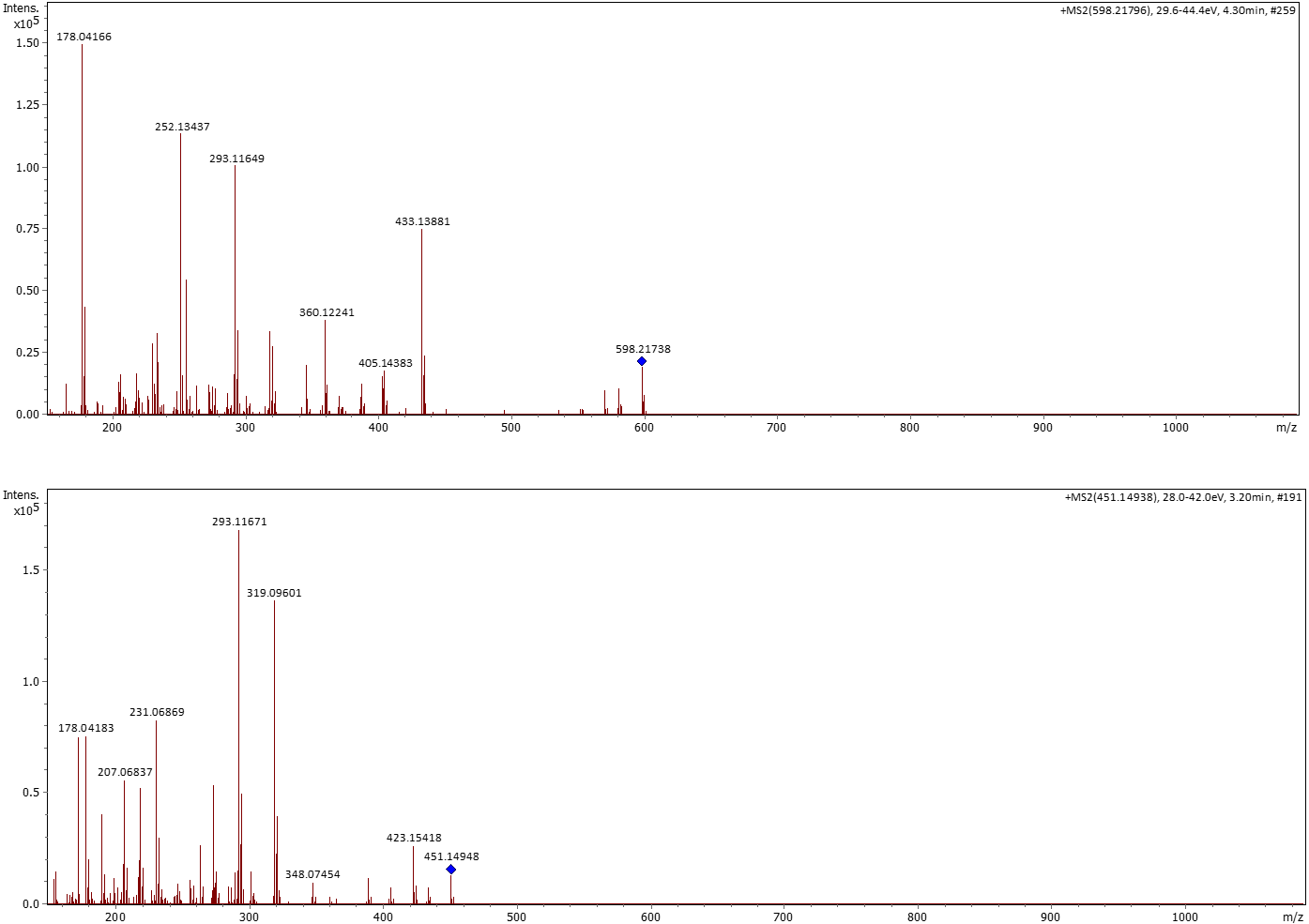


**Figure S23.** LC-MS/MS data for the fragments observed after partial acidic hydrolysis (6N HCl, 110 °C, 20 min) of Saarvienin A.

**Figure S24.** Absolute configuration of amino acids determined using Marfey’s method via comparison of the retention times and MS data of the derivatized amino acids (DAPA, HHPP, and Phe) in the hydrolysate (hydrolysis using 6N HCl; 110 °C; 24 h) to those of the derivatized standard amino acids. Extracted-ion chromatograms (LCMS) are shown. Trp degradation and DAPA racemization was observed upon hydrolysis under these conditions.

**Figure S25.** Absolute configuration of (2*S*,3*R*)-*β*-Me-Cl-Trp determined using Marfey’s method. Extracted-ion chromatograms (LCMS) are shown. Peaks for racemized tryptophan derivative are observed at 21.5 min (with l-FDLA) and 19.2 min (with d-FLDA), as expected upon hydrolysis (24 h; 110 °C) using 5N NaOH solution.

**Figure S26.** Absolute configuration of DAPA units determined using Marfey’s method after hydrolysis in DCl/D_2_O (20%) mixture (18 h; 110 °C). Green (disubstituted derivatives) and orange (monosubstituted derivatives) peaks represent extracted-ion chromatograms (LCMS) of the different monoisotopic ions of the FDLA derivatives of DAPA, while red (disubstituted derivatives) and blue chromatograms (monosubstituted derivatives) are the corresponding 1^st^ isotope ions. Presence of only the 1^st^ isotope ion for d-DAPA and a mixture of monoisotopic and 1^st^ isotope ions for l-DAPA residues suggests that d-DAPA is formed from l-DAPA via racemization upon hydrolysis in 6 N HCl.

**Figure S27.** Absolute configuration of eremosamine (4-*epi*-vancosamine) determined via derivatization using *S/R*-2-butanol after hydrolysis in 1 N HCL (4 h; 60 °C). Extracted-ion chromatograms (LCMS) are shown.

**Figure S28.** Absolute configuration of rhamnose determined via derivatization using *S/R*-2-butanol after hydrolysis in 1 N HCL (4 h; 60 °C). Extracted-ion chromatograms (LCMS) are shown. The assignments were also further confirmed by GCMS analysis after silylation of the butyl derivatives.

**Figure S29.** Absolute configuration of digitoxose determined via derivatization using *S/R*-α-methoxy-α-(trifluoromethyl)phenylacetyl chloride (MTPACl) after hydrolysis in 1 N HCL (4 h; 60 °C). Extracted-ion chromatograms (LCMS) are shown.

**Figure S30.** Absolute configuration of rhamnose determined via derivatization using *S/R*-α-Methoxy-α-(trifluoromethyl)phenylacetyl chloride (MTPACl) after hydrolysis in 1 N HCL (4 h; 60 °C). Extracted-ion chromatograms (LCMS) are shown.

S6. List of Tables — NMR, LCMS, bioactivity, and XRD data

**Table S1**. 1D and 2D NMR data of saarvienin A (**1**; 4-5 mg) in CD_3_OD/D_2_O (3:1) mixture (500 MHz).

| **Saarvienin A (4-5 mg)** | | | | |
| --- | --- | --- | --- | --- |
| **Atom-#** | ***δ*_H_ (mult., J)** | ***δ*_C_** | **HMBC (H→#C)** | **Key ROESY correlations** |
| **Rhamnose (Rha)** | | | | |
| 1 | 4.96 (br s) | 102.7 | Rha-2, Rha-3/5, **Ere-4** | Rha-2, **Ere-4**, NMe-Ere-5, NMe-Ere-6/Ere-6 |
| 2 | 3.93 (br t, 2.3) | 81.4 | Rha-3, Rha-4, **NMe-Ere-1** | Rha-1, Rha-3, **NMe-Ere-1** |
| 3 | 3.83 (dd, 9.4, 2.3) | 71.05 | Rha-4 | Rha-2 (wk), Rha-5 |
| 4 | 3.37 (t, 9.4) | 74.4 | Rha-3/5, Rha-6 | Rha-2 (wk), Rha-6, NMe-Ere-7 |
| 5 | 3.65 (dq, 9.4, 6.1) | 71.0 | Rha-1 (wk), Rha-3, Rha-4, Rha-6 | Rha-3, Rha-6, Ere-6 |
| 6 | 1.23 (d, 6.1) | 18.5 | Rha-4, Rha-5 | Rha-4, Rha-5 |
| **Digitoxose (Dig)** | | | | |
| 1 | 5.02 (br t, 3.2) | 98.6 | Dig-3, Dig-5, **NMe-Ere-4** | Dig-2b, **NMe-Ere-4**, NMe-Ere-6 |
| 2a | 2.17 (dd, 13.7, 3.2) | 34.5 | Dig-1, Dig-3, Dig-4 | Dig-2b, Dig-3 (wk), NMe-Ere-4 |
| 2b | 1.96 (m) |  | Dig-1 | Dig-1, Dig-2a, Dig-3 (wk), Dig-4 (wk) |
| 3 | 4.32 (m) | 63.3 | Dig-4, Dig-5 | Dig-2a/b (wk), Dig-4, N,O-Me-Ere-1 |
| 4 | 3.40 (dd, 8.6, 2.4) | 77.9 | Dig-3, Dig-5, Dig-6, **N,O-Me-Ere-1** | Dig-2b (wk), Dig-3, Dig-6, **N,O-Me-Ere-1**, N,O-Me-Ere-5 |
| 5 | 4.29 (dq, 8.6, 6.5) | 66.0 | Dig-1, Dig-3, Dig-4, Dig-6 | Dig-4, Dig-6 |
| 6 | 1.25 (d, 6.5) | 18.8 | Dig-4, Dig-5 | Dig-4, Dig-5, N,O-Me-Ere-5 |
| **Eremosamine (Ere)** | | | | |
| 1 | 5.60 (br d, 3.1) | 95.8 | Ere-3, Ere-5, **HHPP-7** | Ere-2b, **HHPP-6/8** |
| 2a | 2.28 (d, 14.1) | 39.99 | Ere-1, Ere-3, Ere-4, Ere-7 | Ere-2b, Ere-7 (wk) |
| 2b | 2.20 (dd, 14.1, 3.1) |  | Ere-1, Ere-3, Ere-7 | Ere-1, Ere-2b, Ere-4 (wk) |
| 3 |  | 57.1 |  |  |
| 4 | 3.51 (d, 8.9) | 86.8 | Ere-3, Ere-5, Ere-6, Ere-7, **Rha-1** | Ere-2b, Ere-6, **Rha-1** |
| 5 | 3.88 (dq, 8.9, 6.3) | 67.5 | Ere-3, Ere-4, Ere-5 | Ere-6, Ere-7 |
| 6 | 1.34 (d, 6.3) | 19.3 | Ere-4, Ere-5 | Ere-4, Ere-5 |
| 7 | 1.59 (s) | 20.6 | Ere-2, Ere-3, Ere-4 | Ere-2a (wk), Ere-5 |
| ***N*-methyl-eremosamine (NMe-Ere)** | | | | |
| 1 | 5.14 (br d, 3.6) | 99.9 | NMe-Ere-3, NMe-Ere-5, **Rha-2** | NMe-Ere-2b, **Rha-2** |
| 2a | 2.43 (d, 13.1) | 37.4 | NMe-Ere-1, NMe-Ere-3, NMe-Ere-4, NMe-Ere-7 | NMe-Ere-2b, NMe-Ere-7, NMe-Ere-8 (wk) |
| 2b | 1.92 (m) |  | NMe-Ere-1, NMe-Ere-3, NMe-Ere-7 | NMe-Ere-1, NMe-Ere-2a |
| 3 |  | 61.95 |  |  |
| 4 | 3.44 (d, 9.2) | 80.2 | NMe-Ere-3, NMe-Ere-5, NMe-Ere-6, NMe-Ere-7, **Dig-1** | NMe-Ere-2b, NMe-Ere-6, NMe-Ere-8 (wk), **Dig-1**, Dig-2a |
| 5 | 3.98 (dq, 9.2, 6.2) | 67.3 | NMe-Ere-3, NMe-Ere-4, NMe-Ere-6 | NMe-Ere-6, NMe-Ere-7, Rha-1 |
| 6 | 1.36 (d, 6.2) | 18.7 | NMe-Ere-4, NMe-Ere-5 | NMe-Ere-4, NMe-Ere-5, Dig-1 |
| 7 | 1.55 (s) | 17.3 | NMe-Ere-2, NMe-Ere-3, NMe-Ere-4 | NMe-Ere-2a, NMe-Ere-5, NMe-Ere-8, Rha-4 |
| 8 | 2.64 (s) | 26.4 | NMe-Ere-3 | NMe-Ere-2a |
| ***N,O*-dimethyl-eremosamine (N,O-Me-Ere)** | | | | |
| 1 | 5.17 (br d, 4.5) | 94.0 | N,O-Me-Ere-3, N,O-Me-Ere-5, **Dig-4** | N,O-Me-Ere-2b, Dig-3, **Dig-4** |
| 2a | 2.25 (d, 14.4) | 37.3 | N,O-Me-Ere-1, N,O-Me-Ere-3, N,O-Me-Ere-4 | N,O-Me-Ere-2b |
| 2b | 1.94 (m) |  | N,O-Me-Ere-1, N,O-Me-Ere-3 | N,O-Me-Ere-1, N,O-Me-Ere-2a, N,O-Me-Ere-4 |
| 3 |  | 61.86 |  |  |
| 4 | 3.12 (d, 9.4) | 86.0 | N,O-Me-Ere-3, N,O-Me-Ere-5, N,O-Me-Ere-6, N,O-Me-Ere-7 | N,O-Me-Ere-2b, N,O-Me-Ere-5, N,O-Me-Ere-6, N,O-Me-Ere-8, N,O-Me-Ere-9 |
| 5 | 3.85 (m) | 67.3 | N,O-Me-Ere-3, N,O-Me-Ere-4, N,O-Me-Ere-6 | N,O-Me-Ere-4, N,O-Me-Ere-7, Dig-4, Dig-6 |
| 6 | 1.33 (d, 6.0) | 18.5 | N,O-Me-Ere-4, N,O-Me-Ere-5 | N,O-Me-Ere-4, N,O-Me-Ere-5, N,O-Me-Ere-9 |
| 7 | 1.47 (s) | 16.8 | N,O-Me-Ere-2, N,O-Me-Ere-3, N,O-Me-Ere-4 | N,O-Me-Ere-5, N,O-Me-Ere-8 |
| 8 | 2.60 (s) | 26.6 | N,O-Me-Ere-3 | N,O-Me-Ere-4, N,O-Me-Ere-7 |
| 9 | 3.58 (s) | 62.4 | N,O-Me-Ere-4 | N,O-Me-Ere-4, N,O-Me-Ere-6 |
| **Phenylalanine (Phe)** | | | | |
| 1 |  | 177.6 |  |  |
| 2 | 4.35 (m) | 56.8 | **Phe-1**, Phe-3, **DAPA2-1** | Phe-3a |
| 3a | 3.10 (br dd, 11.4, 3.3) | 39.95 | Phe-1, Phe-2, Phe-4, Phe-5/9 | Phe-2, Phe-3b |
| 3b | 2.39 (br t, 3.3) |  |  | Phe-3a |
| 4 |  | 138.6 |  |  |
| 5/9 | 6.61 (br s) | 130.3 | Phe-7 | Phe-2, Phe-3a (wk), Phe-6/8 |
| 6/8 | 6.94 (m) | 129.2 | Phe-4 | Phe-5/9 |
| 7 | 6.94 (m) | 127.4 | Phe-5/9 | Phe-6/8 |
| **2-hydroxy-3-(4-hydroxyphenyl)propanoic acid (HHPP)** | | | | |
| 1 |  | 175.9 |  |  |
| 2 | 4.34 (m) | 73.7 | HHPP-1, HHPP-3, HHPP-4 | HHPP-3, HHPP-5/9 |
| 3a/b | 2.98 (m, 2H) | 40.5 | HHPP-1, HHPP-2, HHPP-5 | HHPP-2, HHPP-5/9 |
| 4 |  | 132.2 |  |  |
| 5/9 | 7.14 (br d, 8.1) | 132.0 | HHPP-3, HHPP-4, HHPP-7 | HHPP-2, HHPP-3, HHPP-6/8 |
| 6/8 | 6.94 (m) | 117.7 | HHPP-4, HHPP-7 | HHPP-5/9, Ere-1 |
| 7 |  | 156.6 |  |  |
| **(2*S*,3*R*)-*β*-Me-9-Cl-Trp (Trp)** | | | | |
| 1 |  | 174.0 |  |  |
| 2 | 4.84 (ov) | 60.1 | **Trp-1, DAPA1-1** |  |
| 3 | 3.53 (m) | 33.2 | Trp-2, Trp-4, Trp-5, Trp-12 | Trp-5, Trp-11, Trp-12 |
| 4 |  | 118.1 |  |  |
| 5 | 7.37 (s) | 124.1 | Trp-3 (wk), Trp-4, Trp-6, Trp-11 | Trp-2, Trp-3, Trp-12 (wk) |
| 6 |  | 136.1 |  |  |
| 7 | 7.27 (d, 8.6) | 114.0 | Trp-9, Trp-10, Trp-11 | Trp-8 |
| 8 | 7.00 (dd, 8.6, 1.0) | 122.8 | Trp-6, Trp-9, Trp-10 | Trp-7 |
| 9 |  | 125.6 |  |  |
| 10 | 7.60 (br s) | 119.1 | Trp-4 (wk), Trp-6, Trp-8, Trp-9 | Trp-3, Trp-12 (wk) |
| 11 |  | 129.2 |  |  |
| 12 | 1.28 (d, 6.7) | 20.3 | Trp-2, Trp-3, Trp-4 | Trp-3 |
| **2,3-diaminopropionic acid (DAPA1)** | | | | |
| 1 |  | 172.3 |  |  |
| 2 | 4.33 (m) | 54.0 |  |  |
| 3a | 2.94 (m) | 42.3 | **Urea CO, DAPA1-1** | DAPA1-2, DAPA1-3b |
| 3b | 3.79 (m) |  |  | DAPA1-3a |
| **2,3-diaminopropionic acid (DAPA2)** | | | | |
| 1 |  | 170.2 |  |  |
| 2 | 4.25 (m) | 55.2 | **DAPA2-1*, Trp-1*** |  |
| 3a | 3.00 (m) | 42.1 | **Urea CO** | DAPA2-3b |
| 3b | 3.54 (m) |  |  | DAPA2-3a |
| **Urea** | | | | |
| C=O |  | 161.0 |  |  |

*These correlations were observed in the HMBC data of 2 mg sample of saarvienin A (Figure S14).

**Table S2.** Absolute configuration of amino acids determined using Marfey’s method via comparison of the retention times (min) and MS data of the derivatized amino acids in the hydrolysate to those of the derivatized standard amino acids.

| **Amino acid** | **Saarvienin A-l-FDLA** | **Saarvienin A-d-FDLA** | **Amino acid standard-l-FDLA** | **Amino acid standard-d-FDLA** | **Assignment** |
| --- | --- | --- | --- | --- | --- |
| l-Phe | 17.8 | 20.5 | 20.5 | 17.8 | d-Phe |
| l-HHPP | 16.7 | 16.9 | 16.9 | 16.7 | d-HHPP |
| l-DAPA | 8.0, 9.5 (monosubstituted)  21.2 (disubstituted) | 8.7, 9.6 (monosubstituted)  21.5 (disubstituted) | 8.0, 9.5 (monosubstituted)  21.2 (disubstituted) | 8.7, 9.6 (monosubstituted)  21.5 (disubstituted) | l-DAPA |
| (2*S*,3*R*)-*β*-Me-Cl-Trp | 19.6 | 21.3 | 19.6 | 21.3 | (2*S*,3*R*)-*β*-Me-Cl-Trp |

**Table S3.** Absolute configuration of sugars determined via comparison of the retention times (min) and MS data of the *R*/*S*-2-butyl derivatives of sugars in the hydrolysate to those of standard sugars.

| **Sugars** | **Saarvienin A-*R*-Butanol** | **Saarvienin A-*S*-Butanol** | **Sugar-*R*-Butanol** | **Sugar-*S*-Butanol** | **Assignment** |
| --- | --- | --- | --- | --- | --- |
| l-Eremosamine  (4-epi-vancosamine)* | 5.5 | 6.5 | 5.5 | 6.6 | l-Eremosamine |
| l-Rhamnose** | 4.4 | 4.7 | 4.4 | 4.7 | l-Rhamnose |

*l-Eremosamine standard was generated via hydrolysis of eremomycin (Supplier: MedChemExpress).

**Absolute configuration of rhamnose was also confirmed by GCMS analysis after silylation of the butanol derivatives. l-rhamnose-*R*-Butanol: *t*_R_ = 28.5 min; l-rhamnose-*S*-Butanol: *t*_R_ = 28.7 min

**Table S4.** Absolute configuration of sugars determined via comparison of the retention times (min) and MS data of the *R*/*S*-α-methoxy-α-(trifluoromethyl)phenylacetyl (MTPA) derivatives of sugars in the hydrolysate to those of standard sugars.

| **Sugars** | **Saarvienin A-*R*-MTPACl** | **Saarvienin A-*S*-MTPACl** | **Sugar-*S*-MTPACl** | **Assignment** |
| --- | --- | --- | --- | --- |
| d-Digitoxose | 19.0 | 18.0 | 19.1 | l-Digitoxose |
| l-Rhamnose | 11.9, 13.1, 13.4 | 11.6, 12.9, 13.5 | 12.0, 13.2, 13.4 | l-Rhamnose |

**Table S5**. Minimum inhibitory concentrations (MICs) of saarvienin A against a collection of vancomycin-resistant *E. faecium* clinical isolates with reduced susceptibility to daptomycin [n=26]. Values represent the mean of two independent experiments.

| ***Enterococcus faecium* [n=26]** | **MIC [µg/mL]** | | | | | | | |
| --- | --- | --- | --- | --- | --- | --- | --- | --- |
|  | **Saarvienin A** | **Daptomycin** | **Vancomycin** | **Ciprofloxacin** | **Linezolid** | **Gentamicin** | **Ampicillin** | **Teicoplanin** |
| **1** | **1** | **8** | **> 64** | **64** | **2** | **> 64** | **> 64** | **64** |
| **2** | **0.5** | **8** | **> 64** | **> 64** | **2** | **> 64** | **> 64** | **32** |
| **3** | **0.5** | **8** | **64** | **> 64** | **4** | **64** | **64** | **2** |
| **4** | **0.5** | **8** | **> 64** | **> 64** | **2** | **> 64** | **> 64** | **2** |
| **5** | **0.5** | **8** | **> 64** | **> 64** | **4** | **> 64** | **> 64** | **> 64** |
| **6** | **0.5** | **8** | **> 64** | **> 64** | **4** | **> 64** | **> 64** | **64** |
| **7** | **0.5** | **8** | **> 64** | **> 64** | **2** | **> 64** | **> 64** | **32** |
| **8** | **0.5** | **8** | **> 64** | **64** | **2** | **> 64** | **> 64** | **64** |
| **9** | **0.5** | **8** | **> 64** | **> 64** | **2** | **> 64** | **> 64** | **> 64** |
| **10** | **0.5** | **8** | **> 64** | **> 64** | **2** | **> 64** | **> 64** | **> 64** |
| **11** | **0.5** | **8** | **> 64** | **> 64** | **2** | **> 64** | **> 64** | **32** |
| **12** | **0.5** | **8** | **> 64** | **> 64** | **4** | **> 64** | **> 64** | **64** |
| **13** | **0.5** | **8** | **> 64** | **> 64** | **4** | **> 64** | **> 64** | **32** |
| **14** | **0.5** | **8** | **> 64** | **> 64** | **4** | **> 64** | **> 64** | **32** |
| **15** | **0.5** | **8** | **> 64** | **> 64** | **4** | **> 64** | **> 64** | **64** |
| **16** | **0.5** | **8** | **> 64** | **8** | **4** | **> 64** | **> 64** | **32** |
| **17** | **0.5** | **8** | **> 64** | **64** | **2** | **> 64** | **> 64** | **64** |
| **18** | **0.5** | **8** | **> 64** | **> 64** | **4** | **> 64** | **> 64** | **32** |
| **19** | **0.5** | **8** | **> 64** | **> 64** | **4** | **> 64** | **> 64** | **64** |
| **20** | **0.5** | **8** | **> 64** | **32** | **2** | **> 64** | **> 64** | **64** |
| **21** | **0.5** | **8** | **> 64** | **32** | **2** | **> 64** | **> 64** | **> 64** |
| **22** | **0.5** | **8** | **> 64** | **> 64** | **4** | **> 64** | **> 64** | **64** |
| **23** | **0.5** | **8** | **> 64** | **> 64** | **4** | **> 64** | **> 64** | **> 64** |
| **24** | **0.5** | **16** | **> 64** | **> 64** | **4** | **> 64** | **> 64** | **> 64** |
| **25** | **0.5** | **16** | **> 64** | **> 64** | **4** | **> 64** | **> 64** | **> 64** |
| **26** | **0.5** | **8** | **> 64** | **64** | **2** | **> 64** | **> 64** | **> 64** |
| ***E. faecalis* ATCC29212** | **2** | **4** | **4** | **2** | **2** | **8** | **2** | **0.5** |

**Table S6.** Genes in the predicted saarvienin A biosynthesis gene cluster and their putative products and functions.

| **Gene** | **Putative gene product** | **Putative function in saarvienin biosynthesis** |
| --- | --- | --- |
| ctg2_4498 | glucose-1-phosphate adenylyl/thymidylyltransferase | deoxysugar biosynthesis |
| ctg2_4499 | bacterial pleckstrin homology domain protein | resistance |
| ctg2_4500 | LmbU family transcriptional regulator | regulation |
| ctg2_4501 | electron transfer flavoprotein FAD-binding domain | unknown |
| ctg2_4502 | sensor histidine kinase | unknown |
| ctg2_4503 | bacterial pleckstrin homology domain protein | resistance |
| ctg2_4504 | acetyltransferase (GNAT) domain protein | unknown |
| ctg2_4505 | AMP-binding protein | activation of 2-hydroxy-3-(4-hydroxyphenylpropanoic acid) |
| ctg2_4506 | aromatic amino acid lyase | biosynthesis of 2-hydroxy-3-(4-hydroxyphenylpropanoic acid) |
| ctg2_4507 | ABC transporter | efflux of saarvienin or its precursors |
| ctg2_4508 | O-methyltransferase | O-methylation of eremosamine |
| ctg2_4509 | glycosyltransferase, MGT family | glycosylation of the peptide core |
| ctg2_4510 | activator-dependent family glycosyltransferase | glycosylation of the peptide core |
| ctg2_4511 | sodium/hydrogen exchanger family transporter | efflux of saarvienin or its precursors |
| ctg2_4512 | flavin reductase domain protein FMN-binding | unknown |
| ctg2_4513 | tryptophan halogenase | chlorination of tryptophan |
| ctg2_4514 | C-methyltransferase | methylation of tryptophan |
| ctg2_4515 | activator-dependent family glycosyltransferase | glycosylation of the peptide core |
| ctg2_4516 | putative GDP-mannose 4,6 dehydratase | deoxysugar biosynthesis |
| ctg2_4517 | FAD-dependent oxidoreductase | unknown |
| ctg2_4518 | activator-dependent family glycosyltransferase | glycosylation of the peptide core |
| ctg2_4519 | putative N-methyltransferase | N-methylation of eremosamine |
| ctg2_4520 | short chain dehydrogenase | unknown |
| ctg2_4521 | oxidoreductase family, NAD-binding Rossmann fold | unknown |
| ctg2_4522 | dTDP-4-dehydrorhamnose 3,5-epimerase | eremosamine biosynthesis |
| ctg2_4523 | NDP-hexose 2,3-dehydratase | eremosamine biosynthesis |
| ctg2_4524 | DegT/DnrJ/EryC1/StrS aminotransferase | eremosamine biosynthesis |
| ctg2_4525 | GNAT family N-acetyltransferase | unknown |
| ctg2_4526 | hypothetical protein | unknown |
| ctg2_4527 | 2,3-diaminopropionate biosynthesis protein A | l-DAPA biosynthesis |
| ctg2_4528 | 2,3-diaminopropionate biosynthesis protein B | l-DAPA biosynthesis |
| ctg2_4529 | MbtH family NRPS accessory protein | peptide core biosynthesis |
| ctg2_4530 | NRPS: 4 modules | peptide core biosynthesis |
| ctg2_4531 | NRPS: 2 modules-TE | peptide core biosynthesis |
| ctg2_4532 | aspartate/ornithine carbamoyltransferase | unknown |
| ctg2_4533 | LuxR family regulator | regulation |

**Table S7**. Crystal data and structure refinement for (2*S*,3*R*)-*β*-Me-Cl-Trp.

| Identification code | (2*S*,3*R*)-*β*-Me-Cl-Trp (**CCDC #: 2412062**)^[9]^ |  |
| --- | --- | --- |
| Empirical formula | C12 H16.28 Cl2 N2 O3.14 |  |
| Formula weight | 309.7 |  |
| Temperature | 130(2) K |  |
| Wavelength | 0.71073 Å |  |
| Crystal system | Monoclinic |  |
| Space group | P2**_1_** |  |
| Unit cell dimensions | a = 18.8244(7) Å | α= 90°. |
|  | b = 6.7204(2) Å | β= 118.853(5)°. |
|  | c = 19.3129(7) Å | γ = 90°. |
| Volume | 2139.93(16) Å^3^ |  |
| Z | 6 |  |
| Density (calculated) | 1.442 Mg/m^3^ |  |
| Absorption coefficient | 0.461 mm^-1^ |  |
| F(000) | 968 |  |
| Crystal size | 0.120 x 0.080 x 0.020 mm^3^ |  |
| Theta range for data collection | 2.408 to 25.681°. |  |
| Index ranges | -22<=h<=22, -8<=k<=8, -23<=l<=23 |  |
| Reflections collected | 32426 |  |
| Independent reflections | 7983 [R(int) = 0.0436] |  |
| Completeness to theta = 25.242° | 99.90% |  |
| Absorption correction | Semi-empirical from equivalents |  |
| Max. and min. transmission | 1.0000 and 0.9627 |  |
| Refinement method | Full-matrix least-squares on F^2^ |  |
| Data / restraints / parameters | 7983 / 40 / 596 |  |
| Goodness-of-fit on F2 | 1.028 |  |
| Final R indices [I>2sigma(I)] | R1 = 0.0499, wR2 = 0.1198 |  |
| R indices (all data) | R1 = 0.0544, wR2 = 0.1224 |  |
| Absolute structure parameter | 0.01(2) |  |
| Extinction coefficient | n/a |  |
| Largest diff. peak and hole | 1.623 and -0.825 e.Å^-3^ |  |

**Table S8**. Atomic coordinates (x 104) and equivalent isotropic displacement parameters (Å2x 103) for (2*S*,3*R*)-*β*-Me-Cl-Trp. U(eq) is defined as one third of the trace of the orthogonalized Uij tensor.

|  | **x** | **y** | **z** | **U(eq)** |
| --- | --- | --- | --- | --- |
| Cl(4) | 7951(1) | -567(2) | 7374(1) | 29(1) |
| Cl(5) | 5766(1) | -1978(2) | 8000(1) | 35(1) |
| Cl(6) | 2829(1) | 6195(2) | 5589(1) | 41(1) |
| Cl(1) | 8243(1) | 3067(3) | 2853(1) | 55(1) |
| O(1) | 7372(3) | 4441(6) | 6541(2) | 40(1) |
| O(2) | 8257(3) | 2463(6) | 6409(2) | 30(1) |
| N(1) | 7494(3) | 7381(7) | 5668(3) | 25(1) |
| N(2) | 9343(3) | 9885(8) | 4997(4) | 42(1) |
| C(1) | 7810(3) | 4108(8) | 6266(3) | 27(1) |
| C(2) | 7940(3) | 5495(8) | 5716(3) | 23(1) |
| C(3) | 8844(3) | 5861(8) | 5989(3) | 26(1) |
| C(4) | 8970(3) | 7227(8) | 5449(3) | 25(1) |
| C(5) | 9254(4) | 9149(9) | 5607(4) | 37(1) |
| C(6) | 9112(3) | 8447(9) | 4415(4) | 35(1) |
| C(7) | 9136(4) | 8465(11) | 3708(4) | 46(2) |
| C(8) | 8873(4) | 6789(13) | 3248(4) | 48(2) |
| C(9) | 8602(4) | 5110(10) | 3490(3) | 37(2) |
| C(10) | 8587(3) | 5058(8) | 4194(3) | 25(1) |
| C(11) | 8861(3) | 6756(8) | 4673(3) | 25(1) |
| C(12) | 9309(4) | 6554(10) | 6857(3) | 35(1) |
| Cl(2) | 2835(1) | 5417(2) | -478(1) | 39(1) |
| O(3) | 6340(3) | 6334(7) | 4063(2) | 53(1) |
| O(4) | 6350(2) | 4325(6) | 3138(2) | 37(1) |
| N(3) | 5531(3) | 9235(7) | 3053(3) | 27(1) |
| N(4) | 5136(3) | 11975(7) | 758(3) | 30(1) |
| C(13) | 6152(3) | 5958(8) | 3386(3) | 32(1) |
| C(14) | 5652(3) | 7374(8) | 2709(3) | 24(1) |
| C(15) | 6031(3) | 7788(8) | 2165(3) | 27(1) |
| C(16) | 5528(3) | 9234(8) | 1518(3) | 24(1) |
| C(17) | 5730(3) | 11133(9) | 1440(3) | 29(1) |
| C(18) | 4531(3) | 10622(9) | 377(3) | 26(1) |
| C(19) | 3822(3) | 10749(9) | -356(3) | 28(1) |
| C(20) | 3313(3) | 9137(10) | -604(3) | 30(1) |
| C(21) | 3510(3) | 7404(9) | -138(3) | 28(1) |
| C(22) | 4218(3) | 7240(8) | 582(3) | 24(1) |
| C(23) | 4744(3) | 8850(8) | 841(3) | 21(1) |
| C(24) | 6912(3) | 8470(10) | 2638(4) | 40(2) |
| Cl(3) | 10299(1) | 641(2) | 8096(1) | 28(1) |
| O(5) | 6146(2) | 3065(6) | 7830(3) | 36(1) |
| O(6) | 7133(2) | 983(5) | 8626(2) | 26(1) |
| N(5) | 7125(3) | 5780(7) | 7786(3) | 24(1) |
| N(6) | 9775(3) | 8038(7) | 9359(3) | 24(1) |
| C(25) | 6851(3) | 2629(7) | 8211(3) | 21(1) |
| C(26) | 7519(3) | 3986(7) | 8262(3) | 18(1) |
| C(27) | 8149(3) | 4519(8) | 9119(3) | 21(1) |
| C(28) | 8847(3) | 5660(8) | 9137(3) | 21(1) |
| C(29) | 9082(3) | 7549(8) | 9398(3) | 22(1) |
| C(30) | 9997(3) | 6440(8) | 9068(3) | 20(1) |
| C(31) | 10678(3) | 6163(8) | 8965(3) | 21(1) |
| C(32) | 10759(3) | 4364(8) | 8674(3) | 22(1) |
| C(33) | 10176(3) | 2862(8) | 8489(3) | 20(1) |
| C(34) | 9510(3) | 3073(8) | 8605(3) | 21(1) |
| C(35) | 9422(3) | 4912(7) | 8904(3) | 18(1) |
| C(36) | 7760(3) | 5576(10) | 9550(3) | 28(1) |
| O(1W) | 4675(4) | 8795(9) | 3970(4) | 73(2) |
| O(2W) | 5883(3) | 6937(12) | 5364(4) | 89(2) |
| O(3W) | 3950(6) | 5080(20) | 3490(5) | 172(6) |
| O(4W) | 4033(9) | 7580(20) | 5158(8) | 66(5) |

**Table S9**. Bond lengths [Å] and angles [°] for (2*S*,3*R*)-*β*-Me-Cl-Trp.

| Cl(1)-C(9) | 1.748(7) | C(11)-C(4)-C(3) | 126.8(5) |
| --- | --- | --- | --- |
| O(1)-C(1) | 1.197(7) | N(2)-C(5)-C(4) | 110.2(6) |
| O(2)-C(1) | 1.335(7) | N(2)-C(6)-C(7) | 130.3(6) |
| N(1)-C(2) | 1.499(7) | N(2)-C(6)-C(11) | 107.6(5) |
| N(2)-C(5) | 1.360(9) | C(7)-C(6)-C(11) | 122.0(6) |
| N(2)-C(6) | 1.384(9) | C(8)-C(7)-C(6) | 117.3(6) |
| C(1)-C(2) | 1.519(7) | C(7)-C(8)-C(9) | 121.2(6) |
| C(2)-C(3) | 1.538(7) | C(10)-C(9)-C(8) | 122.2(6) |
| C(3)-C(4) | 1.493(8) | C(10)-C(9)-Cl(1) | 119.4(5) |
| C(3)-C(12) | 1.541(7) | C(8)-C(9)-Cl(1) | 118.4(5) |
| C(4)-C(5) | 1.375(8) | C(9)-C(10)-C(11) | 117.1(5) |
| C(4)-C(11) | 1.445(8) | C(10)-C(11)-C(6) | 120.2(5) |
| C(6)-C(7) | 1.387(9) | C(10)-C(11)-C(4) | 133.2(5) |
| C(6)-C(11) | 1.411(8) | C(6)-C(11)-C(4) | 106.6(5) |
| C(7)-C(8) | 1.371(11) | C(18)-N(4)-C(17) | 108.9(5) |
| C(8)-C(9) | 1.408(10) | O(3)-C(13)-O(4) | 125.5(5) |
| C(9)-C(10) | 1.375(8) | O(3)-C(13)-C(14) | 122.5(5) |
| C(10)-C(11) | 1.401(8) | O(4)-C(13)-C(14) | 112.0(5) |
| Cl(2)-C(21) | 1.739(6) | N(3)-C(14)-C(13) | 107.9(4) |
| O(3)-C(13) | 1.205(7) | N(3)-C(14)-C(15) | 111.8(4) |
| O(4)-C(13) | 1.321(7) | C(13)-C(14)-C(15) | 113.4(5) |
| N(3)-C(14) | 1.484(7) | C(16)-C(15)-C(24) | 111.6(5) |
| N(4)-C(18) | 1.363(7) | C(16)-C(15)-C(14) | 111.6(4) |
| N(4)-C(17) | 1.372(7) | C(24)-C(15)-C(14) | 111.7(5) |
| C(13)-C(14) | 1.521(7) | C(17)-C(16)-C(23) | 106.6(5) |
| C(14)-C(15) | 1.553(8) | C(17)-C(16)-C(15) | 127.0(5) |
| C(15)-C(16) | 1.504(7) | C(23)-C(16)-C(15) | 126.4(5) |
| C(15)-C(24) | 1.528(7) | C(16)-C(17)-N(4) | 110.6(5) |
| C(16)-C(17) | 1.360(8) | N(4)-C(18)-C(19) | 130.1(5) |
| C(16)-C(23) | 1.447(7) | N(4)-C(18)-C(23) | 108.0(4) |
| C(18)-C(19) | 1.403(7) | C(19)-C(18)-C(23) | 121.8(5) |
| C(18)-C(23) | 1.427(7) | C(20)-C(19)-C(18) | 118.0(5) |
| C(19)-C(20) | 1.370(9) | C(19)-C(20)-C(21) | 120.4(5) |
| C(20)-C(21) | 1.408(8) | C(22)-C(21)-C(20) | 122.3(5) |
| C(21)-C(22) | 1.390(7) | C(22)-C(21)-Cl(2) | 119.4(5) |
| C(22)-C(23) | 1.386(7) | C(20)-C(21)-Cl(2) | 118.3(4) |
| Cl(3)-C(33) | 1.740(5) | C(23)-C(22)-C(21) | 118.1(5) |
| O(5)-C(25) | 1.202(6) | C(22)-C(23)-C(18) | 119.3(5) |
| O(6)-C(25) | 1.318(6) | C(22)-C(23)-C(16) | 134.8(5) |
| N(5)-C(26) | 1.480(6) | C(18)-C(23)-C(16) | 105.8(5) |
| N(6)-C(30) | 1.368(7) | C(30)-N(6)-C(29) | 108.6(4) |
| N(6)-C(29) | 1.383(7) | O(5)-C(25)-O(6) | 125.4(5) |
| C(25)-C(26) | 1.518(7) | O(5)-C(25)-C(26) | 121.9(5) |
| C(26)-C(27) | 1.543(7) | O(6)-C(25)-C(26) | 112.7(4) |
| C(27)-C(28) | 1.506(7) | N(5)-C(26)-C(25) | 107.3(4) |
| C(27)-C(36) | 1.525(7) | N(5)-C(26)-C(27) | 111.9(4) |
| C(28)-C(29) | 1.359(7) | C(25)-C(26)-C(27) | 113.1(4) |
| C(28)-C(35) | 1.448(7) | C(28)-C(27)-C(36) | 113.2(4) |
| C(30)-C(31) | 1.400(7) | C(28)-C(27)-C(26) | 110.7(4) |
| C(30)-C(35) | 1.413(7) | C(36)-C(27)-C(26) | 111.8(4) |
| C(31)-C(32) | 1.372(8) | C(29)-C(28)-C(35) | 106.3(4) |
| C(32)-C(33) | 1.405(7) | C(29)-C(28)-C(27) | 127.4(5) |
| C(33)-C(34) | 1.383(7) | C(35)-C(28)-C(27) | 126.2(5) |
| C(34)-C(35) | 1.408(7) | C(28)-C(29)-N(6) | 110.4(5) |
| C(5)-N(2)-C(6) | 109.2(5) | N(6)-C(30)-C(31) | 130.2(5) |
| O(1)-C(1)-O(2) | 124.3(5) | N(6)-C(30)-C(35) | 108.0(4) |
| O(1)-C(1)-C(2) | 124.9(5) | C(31)-C(30)-C(35) | 121.8(5) |
| O(2)-C(1)-C(2) | 110.8(5) | C(32)-C(31)-C(30) | 117.8(5) |
| N(1)-C(2)-C(1) | 106.7(4) | C(31)-C(32)-C(33) | 120.6(4) |
| N(1)-C(2)-C(3) | 112.4(4) | C(34)-C(33)-C(32) | 122.9(5) |
| C(1)-C(2)-C(3) | 112.5(4) | C(34)-C(33)-Cl(3) | 118.7(4) |
| C(4)-C(3)-C(2) | 112.4(4) | C(32)-C(33)-Cl(3) | 118.3(4) |
| C(4)-C(3)-C(12) | 112.0(5) | C(33)-C(34)-C(35) | 116.8(5) |
| C(2)-C(3)-C(12) | 112.1(4) | C(34)-C(35)-C(30) | 120.1(4) |
| C(5)-C(4)-C(11) | 106.4(5) | C(34)-C(35)-C(28) | 133.3(5) |
| C(5)-C(4)-C(3) | 126.7(5) | C(30)-C(35)-C(28) | 106.6(4) |

**Table S10**. Anisotropic displacement parameters (Å2x 103) for (2*S*,3*R*)-*β*-Me-Cl-Trp. The anisotropic displacement factor exponent takes the form: -2π2[h2 a*2U11 + ... + 2 h k a* b* U12]

|  | **U11** | **U22** | **U33** | **U23** | **U13** | **U12** |
| --- | --- | --- | --- | --- | --- | --- |
| Cl(4) | 45(1) | 18(1) | 28(1) | -1(1) | 20(1) | -5(1) |
| Cl(5) | 25(1) | 20(1) | 54(1) | -9(1) | 14(1) | -2(1) |
| Cl(6) | 46(1) | 18(1) | 29(1) | -2(1) | -6(1) | 6(1) |
| Cl(1) | 64(1) | 70(1) | 26(1) | -2(1) | 18(1) | 27(1) |
| O(1) | 76(3) | 25(2) | 39(2) | 7(2) | 43(2) | 11(2) |
| O(2) | 48(2) | 17(2) | 27(2) | 9(2) | 18(2) | 9(2) |
| N(1) | 33(2) | 20(2) | 18(2) | -1(2) | 10(2) | -1(2) |
| N(2) | 29(3) | 21(3) | 68(4) | 14(3) | 19(3) | -2(2) |
| C(1) | 37(3) | 20(3) | 18(2) | -3(2) | 8(2) | 0(2) |
| C(2) | 32(3) | 14(2) | 15(2) | -1(2) | 6(2) | -1(2) |
| C(3) | 31(3) | 22(3) | 21(2) | -1(2) | 9(2) | 4(2) |
| C(4) | 23(3) | 19(3) | 25(3) | 0(2) | 6(2) | 1(2) |
| C(5) | 31(3) | 26(3) | 43(3) | 0(3) | 8(3) | -4(2) |
| C(6) | 21(3) | 35(3) | 47(4) | 18(3) | 16(3) | 7(2) |
| C(7) | 38(3) | 48(4) | 62(4) | 34(4) | 33(3) | 17(3) |
| C(8) | 37(3) | 80(6) | 39(4) | 31(4) | 29(3) | 30(4) |
| C(9) | 35(3) | 49(4) | 27(3) | 8(3) | 15(3) | 20(3) |
| C(10) | 26(3) | 27(3) | 21(2) | 5(2) | 10(2) | 3(2) |
| C(11) | 22(3) | 25(3) | 29(3) | 9(2) | 12(2) | 5(2) |
| C(12) | 33(3) | 37(3) | 22(3) | -4(2) | 3(2) | 8(3) |
| Cl(2) | 26(1) | 42(1) | 41(1) | -14(1) | 10(1) | -10(1) |
| O(3) | 78(3) | 25(2) | 22(2) | 3(2) | -2(2) | 9(2) |
| O(4) | 36(2) | 23(2) | 32(2) | 6(2) | 1(2) | 11(2) |
| N(3) | 22(2) | 18(2) | 29(2) | 3(2) | 4(2) | 3(2) |
| N(4) | 35(3) | 21(2) | 32(3) | 2(2) | 16(2) | -1(2) |
| C(13) | 31(3) | 14(3) | 28(3) | 3(2) | -4(2) | -4(2) |
| C(14) | 20(2) | 17(2) | 23(3) | 0(2) | 1(2) | 0(2) |
| C(15) | 22(2) | 23(3) | 27(3) | 2(2) | 5(2) | 3(2) |
| C(16) | 21(2) | 23(3) | 25(3) | 4(2) | 8(2) | 3(2) |
| C(17) | 25(3) | 26(3) | 33(3) | -2(2) | 11(2) | -2(2) |
| C(18) | 26(3) | 23(3) | 28(3) | 7(2) | 13(2) | 6(2) |
| C(19) | 31(3) | 30(3) | 24(2) | 9(2) | 13(2) | 14(2) |
| C(20) | 21(3) | 47(4) | 21(3) | 1(3) | 8(2) | 11(2) |
| C(21) | 20(2) | 33(3) | 29(3) | -8(2) | 11(2) | -1(2) |
| C(22) | 23(2) | 27(3) | 23(3) | 3(2) | 12(2) | 2(2) |
| C(23) | 17(2) | 21(3) | 22(2) | 2(2) | 7(2) | 5(2) |
| C(24) | 19(3) | 44(4) | 45(4) | 10(3) | 7(3) | 3(2) |
| Cl(3) | 34(1) | 21(1) | 38(1) | -4(1) | 24(1) | 0(1) |
| O(5) | 21(2) | 27(2) | 51(2) | 6(2) | 9(2) | 0(2) |
| O(6) | 26(2) | 16(2) | 37(2) | 4(2) | 16(2) | -2(2) |
| N(5) | 29(2) | 20(2) | 23(2) | 2(2) | 13(2) | 2(2) |
| N(6) | 27(2) | 18(2) | 29(2) | -6(2) | 14(2) | -6(2) |
| C(25) | 26(3) | 15(2) | 25(2) | -3(2) | 16(2) | 0(2) |
| C(26) | 22(2) | 13(2) | 21(2) | 0(2) | 12(2) | -2(2) |
| C(27) | 25(2) | 18(2) | 20(2) | 2(2) | 11(2) | 0(2) |
| C(28) | 22(2) | 19(2) | 20(2) | -3(2) | 9(2) | 0(2) |
| C(29) | 24(2) | 19(2) | 24(2) | 1(2) | 12(2) | -2(2) |
| C(30) | 22(2) | 19(2) | 16(2) | -2(2) | 7(2) | -4(2) |
| C(31) | 19(2) | 21(3) | 21(2) | 1(2) | 7(2) | -5(2) |
| C(32) | 19(2) | 27(3) | 21(2) | 6(2) | 11(2) | 1(2) |
| C(33) | 22(2) | 16(2) | 21(2) | -3(2) | 9(2) | 1(2) |
| C(34) | 23(2) | 18(2) | 18(2) | 2(2) | 7(2) | -2(2) |
| C(35) | 19(2) | 16(2) | 16(2) | 1(2) | 6(2) | -2(2) |
| C(36) | 27(3) | 38(3) | 23(2) | -4(2) | 14(2) | -4(2) |
| O(1W) | 108(5) | 51(4) | 55(3) | -8(3) | 36(3) | -17(3) |
| O(2W) | 47(3) | 116(6) | 76(4) | 47(4) | 7(3) | -21(3) |
| O(3W) | 138(7) | 232(12) | 75(5) | 58(6) | -5(5) | -129(8) |
| O(4W) | 73(10) | 80(11) | 52(8) | -5(7) | 37(7) | -5(8) |

**Table S11**. Hydrogen coordinates (x 104) and isotropic displacement parameters (Å2x 10 3) for (2*S*,3*R*)-*β*-Me-Cl-Trp.

|  | x | y | z | U(eq) |
| --- | --- | --- | --- | --- |
| H(2O) | 8220(40) | 1730(90) | 6740(30) | 46 |
| H(1AN) | 6976(13) | 7180(100) | 5490(40) | 37 |
| H(1BN) | 7600(40) | 8070(90) | 5340(30) | 37 |
| H(1CN) | 7720(40) | 7930(100) | 6141(19) | 37 |
| H(2N) | 9540(40) | 11010(60) | 4930(40) | 50 |
| H(2) | 7687 | 4877 | 5178 | 27 |
| H(3) | 9080 | 4546 | 5965 | 31 |
| H(5) | 9372 | 9861 | 6075 | 45 |
| H(7) | 9327 | 9592 | 3549 | 55 |
| H(8) | 8874 | 6760 | 2756 | 57 |
| H(10) | 8399 | 3920 | 4350 | 30 |
| H(12A) | 9889 | 6640 | 7023 | 52 |
| H(12B) | 9225 | 5598 | 7193 | 52 |
| H(12C) | 9110 | 7865 | 6905 | 52 |
| H(4O) | 6620(40) | 3540(90) | 3510(30) | 55 |
| H(3AN) | 5270(30) | 9500(90) | 3310(30) | 40 |
| H(3BN) | 5260(40) | 10180(70) | 2710(30) | 40 |
| H(3CN) | 6024(18) | 9710(100) | 3340(30) | 40 |
| H(4N) | 5100(40) | 13150(50) | 550(30) | 35 |
| H(14) | 5107 | 6754 | 2378 | 29 |
| H(15) | 6031 | 6500 | 1906 | 32 |
| H(17) | 6214 | 11786 | 1807 | 35 |
| H(19) | 3699 | 11917 | -670 | 34 |
| H(20) | 2824 | 9190 | -1094 | 36 |
| H(22) | 4338 | 6058 | 888 | 29 |
| H(24A) | 7151 | 8560 | 2287 | 60 |
| H(24B) | 7219 | 7508 | 3060 | 60 |
| H(24C) | 6932 | 9778 | 2871 | 60 |
| H(6O) | 6710(20) | 290(90) | 8430(40) | 39 |
| H(5AN) | 6740(30) | 6290(90) | 7870(40) | 36 |
| H(5BN) | 6830(30) | 5090(90) | 7350(20) | 36 |
| H(5CN) | 7470(30) | 6720(70) | 7830(40) | 36 |
| H(6N) | 10000(30) | 9220(40) | 9470(30) | 29 |
| H(26) | 7807 | 3284 | 8013 | 21 |
| H(27) | 8375 | 3236 | 9403 | 25 |
| H(29) | 8808 | 8411 | 9581 | 26 |
| H(31) | 11070 | 7185 | 9092 | 26 |
| H(32) | 11214 | 4133 | 8597 | 26 |
| H(34) | 9130 | 2026 | 8488 | 25 |
| H(36A) | 8162 | 5739 | 10109 | 43 |
| H(36B) | 7304 | 4782 | 9508 | 43 |
| H(36C) | 7563 | 6886 | 9311 | 43 |
| H(1AW) | 4303(19) | 8390(80) | 3532(11) | 109 |
| H(1BW) | 5010(50) | 7840(90) | 4168(18) | 109 |
| H(2AW) | 5840(20) | 8160(30) | 5438(17) | 133 |
| H(2BW) | 5840(50) | 6380(70) | 5730(30) | 133 |
| H(3AW) | 3500(19) | 4570(190) | 3370(70) | 257 |
| H(3BW) | 3920(80) | 5390(60) | 3050(30) | 257 |
| H(4AW) | 3790(90) | 7600(200) | 5420(90) | 98 |
| H(4BW) | 4180(60) | 8750(70) | 5140(60) | 98 |

**Table S12**. Torsion angles [°] for (2*S*,3*R*)-*β*-Me-Cl-Trp.

| O(1)-C(1)-C(2)-N(1) | 6.0(7) | N(4)-C(18)-C(19)-C(20) | 179.5(5) |
| --- | --- | --- | --- |
| O(2)-C(1)-C(2)-N(1) | -173.5(4) | C(23)-C(18)-C(19)-C(20) | 2.8(8) |
| O(1)-C(1)-C(2)-C(3) | 129.6(6) | C(18)-C(19)-C(20)-C(21) | -1.1(8) |
| O(2)-C(1)-C(2)-C(3) | -49.9(6) | C(19)-C(20)-C(21)-C(22) | -0.2(8) |
| N(1)-C(2)-C(3)-C(4) | -59.4(6) | C(19)-C(20)-C(21)-Cl(2) | 179.3(4) |
| C(1)-C(2)-C(3)-C(4) | -179.9(4) | C(20)-C(21)-C(22)-C(23) | -0.3(8) |
| N(1)-C(2)-C(3)-C(12) | 67.8(6) | Cl(2)-C(21)-C(22)-C(23) | -179.7(4) |
| C(1)-C(2)-C(3)-C(12) | -52.6(6) | C(21)-C(22)-C(23)-C(18) | 1.9(7) |
| C(2)-C(3)-C(4)-C(5) | 109.3(6) | C(21)-C(22)-C(23)-C(16) | -177.3(5) |
| C(12)-C(3)-C(4)-C(5) | -18.0(8) | N(4)-C(18)-C(23)-C(22) | 179.4(5) |
| C(2)-C(3)-C(4)-C(11) | -74.3(7) | C(19)-C(18)-C(23)-C(22) | -3.3(8) |
| C(12)-C(3)-C(4)-C(11) | 158.4(5) | N(4)-C(18)-C(23)-C(16) | -1.2(6) |
| C(6)-N(2)-C(5)-C(4) | 0.3(7) | C(19)-C(18)-C(23)-C(16) | 176.1(5) |
| C(11)-C(4)-C(5)-N(2) | -1.1(6) | C(17)-C(16)-C(23)-C(22) | -179.5(6) |
| C(3)-C(4)-C(5)-N(2) | 175.8(5) | C(15)-C(16)-C(23)-C(22) | 2.2(10) |
| C(5)-N(2)-C(6)-C(7) | -176.2(6) | C(17)-C(16)-C(23)-C(18) | 1.2(6) |
| C(5)-N(2)-C(6)-C(11) | 0.8(6) | C(15)-C(16)-C(23)-C(18) | -177.1(5) |
| N(2)-C(6)-C(7)-C(8) | 179.2(6) | O(5)-C(25)-C(26)-N(5) | 0.7(7) |
| C(11)-C(6)-C(7)-C(8) | 2.5(8) | O(6)-C(25)-C(26)-N(5) | -179.1(4) |
| C(6)-C(7)-C(8)-C(9) | -0.9(9) | O(5)-C(25)-C(26)-C(27) | 124.6(5) |
| C(7)-C(8)-C(9)-C(10) | -0.1(9) | O(6)-C(25)-C(26)-C(27) | -55.2(5) |
| C(7)-C(8)-C(9)-Cl(1) | 177.8(5) | N(5)-C(26)-C(27)-C(28) | -65.4(5) |
| C(8)-C(9)-C(10)-C(11) | -0.5(8) | C(25)-C(26)-C(27)-C(28) | 173.3(4) |
| Cl(1)-C(9)-C(10)-C(11) | -178.3(4) | N(5)-C(26)-C(27)-C(36) | 61.8(6) |
| C(9)-C(10)-C(11)-C(6) | 2.0(8) | C(25)-C(26)-C(27)-C(36) | -59.5(6) |
| C(9)-C(10)-C(11)-C(4) | -176.8(6) | C(36)-C(27)-C(28)-C(29) | -8.8(7) |
| N(2)-C(6)-C(11)-C(10) | 179.5(5) | C(26)-C(27)-C(28)-C(29) | 117.6(5) |
| C(7)-C(6)-C(11)-C(10) | -3.2(8) | C(36)-C(27)-C(28)-C(35) | 168.6(5) |
| N(2)-C(6)-C(11)-C(4) | -1.4(6) | C(26)-C(27)-C(28)-C(35) | -65.0(6) |
| C(7)-C(6)-C(11)-C(4) | 175.9(5) | C(35)-C(28)-C(29)-N(6) | -1.0(6) |
| C(5)-C(4)-C(11)-C(10) | -179.6(6) | C(27)-C(28)-C(29)-N(6) | 176.8(5) |
| C(3)-C(4)-C(11)-C(10) | 3.5(10) | C(30)-N(6)-C(29)-C(28) | -0.1(6) |
| C(5)-C(4)-C(11)-C(6) | 1.5(6) | C(29)-N(6)-C(30)-C(31) | -175.6(5) |
| C(3)-C(4)-C(11)-C(6) | -175.4(5) | C(29)-N(6)-C(30)-C(35) | 1.1(5) |
| O(3)-C(13)-C(14)-N(3) | 5.0(8) | N(6)-C(30)-C(31)-C(32) | 178.4(5) |
| O(4)-C(13)-C(14)-N(3) | -174.3(5) | C(35)-C(30)-C(31)-C(32) | 2.0(7) |
| O(3)-C(13)-C(14)-C(15) | 129.5(6) | C(30)-C(31)-C(32)-C(33) | -0.1(7) |
| O(4)-C(13)-C(14)-C(15) | -49.9(6) | C(31)-C(32)-C(33)-C(34) | -1.8(7) |
| N(3)-C(14)-C(15)-C(16) | -56.3(6) | C(31)-C(32)-C(33)-Cl(3) | 178.2(4) |
| C(13)-C(14)-C(15)-C(16) | -178.5(4) | C(32)-C(33)-C(34)-C(35) | 1.6(7) |
| N(3)-C(14)-C(15)-C(24) | 69.5(6) | Cl(3)-C(33)-C(34)-C(35) | -178.4(3) |
| C(13)-C(14)-C(15)-C(24) | -52.7(6) | C(33)-C(34)-C(35)-C(30) | 0.4(7) |
| C(24)-C(15)-C(16)-C(17) | -15.0(8) | C(33)-C(34)-C(35)-C(28) | -176.5(5) |
| C(14)-C(15)-C(16)-C(17) | 110.8(6) | N(6)-C(30)-C(35)-C(34) | -179.3(4) |
| C(24)-C(15)-C(16)-C(23) | 163.0(5) | C(31)-C(30)-C(35)-C(34) | -2.2(7) |
| C(14)-C(15)-C(16)-C(23) | -71.1(7) | N(6)-C(30)-C(35)-C(28) | -1.7(5) |
| C(23)-C(16)-C(17)-N(4) | -0.8(6) | C(31)-C(30)-C(35)-C(28) | 175.4(4) |
| C(15)-C(16)-C(17)-N(4) | 177.5(5) | C(29)-C(28)-C(35)-C(34) | 178.8(5) |
| C(18)-N(4)-C(17)-C(16) | 0.1(6) | C(27)-C(28)-C(35)-C(34) | 0.9(9) |
| C(17)-N(4)-C(18)-C(19) | -176.3(6) | C(29)-C(28)-C(35)-C(30) | 1.6(5) |
| C(17)-N(4)-C(18)-C(23) | 0.7(6) | C(27)-C(28)-C(35)-C(30) | -176.2(4) |

**Table S13**. Hydrogen bonds for (2*S*,3*R*)-*β*-Me-Cl-Trp [Å and °].

| **D-H...A** | **d(D-H)** | **d(H...A)** | **d(D...A)** | **<(DHA)** |
| --- | --- | --- | --- | --- |
| O(2)-H(2O)...Cl(4) | 0.841(14) | 2.17(2) | 2.998(4) | 167(7) |
| O(4)-H(4O)...Cl(6)#1 | 0.841(14) | 2.196(18) | 3.032(4) | 172(8) |
| O(6)-H(6O)...Cl(5) | 0.841(14) | 2.18(2) | 3.007(4) | 169(7) |
| N(1)-H(1AN)...O(2W) | 0.875(14) | 1.96(2) | 2.813(8) | 166(6) |
| N(1)-H(1BN)...Cl(6)#2 | 0.881(14) | 2.63(4) | 3.378(5) | 144(6) |
| N(1)-H(1CN)...Cl(4)#3 | 0.882(14) | 2.43(2) | 3.281(5) | 163(6) |
| N(3)-H(3AN)...O(1W) | 0.869(14) | 2.12(3) | 2.930(9) | 155(6) |
| N(3)-H(3BN)...Cl(5)#4 | 0.879(14) | 2.59(3) | 3.433(5) | 160(6) |
| N(3)-H(3CN)...Cl(6)#2 | 0.880(14) | 2.37(3) | 3.213(5) | 161(6) |
| N(5)-H(5AN)...Cl(5)#3 | 0.877(14) | 2.293(18) | 3.165(5) | 172(6) |
| N(5)-H(5BN)...O(1) | 0.879(14) | 2.29(6) | 2.808(6) | 118(5) |
| N(5)-H(5CN)...Cl(4)#3 | 0.881(14) | 2.38(3) | 3.205(5) | 155(6) |
| O(1W)-H(1AW)...O(5)#2 | 0.841(13) | 2.35(2) | 3.087(7) | 146(3) |
| O(1W)-H(1BW)...O(2W) | 0.847(14) | 2.18(2) | 2.840(9) | 135(3) |
| O(2W)-H(2AW)...O(3W)#2 | 0.848(14) | 2.31(2) | 2.967(12) | 135(3) |
| O(2W)-H(2BW)...O(1W)#1 | 0.842(14) | 2.19(3) | 2.919(9) | 144(4) |
| O(3W)-H(3AW)...Cl(4)#2 | 0.840(14) | 2.40(3) | 3.165(10) | 153(6) |
| O(3W)-H(3BW)...O(5)#2 | 0.845(14) | 2.44(2) | 3.177(9) | 146(4) |
| O(4W)-H(4AW)...Cl(6) | 0.840(14) | 2.21(3) | 2.918(14) | 142(5) |
| O(4W)-H(4BW)...O(2W)#2 | 0.845(14) | 2.33(3) | 3.128(18) | 158(9) |

Symmetry transformations used to generate equivalent atoms:

#1 -x+1,y-1/2,-z+1 #2 -x+1,y+1/2,-z+1 #3 x,y+1,z

#4 -x+1,y+3/2,-z+1

# References

[1] O. N. Sekurova, M. Zehl, M. Predl, P. Hunyadi, T. Rattei, S. B. Zotchev, *Microb. Cell Fact.* **2024**, *23*, 201.

[2] J. F. Guerrero-Garzón, E. Madland, M. Zehl, M. Singh, S. Rezaei, F. L. Aachmann, G. Courtade, E. Urban, C. Rückert, T. Busche et al., *iScience* **2020**, *23*, 101785.

[3] M. Xu, W. Wang, N. Waglechner, E. J. Culp, A. K. Guitor, G. D. Wright, *Nat. Commun.* **2020**, *11*, 5232.

[4] G. M. Sheldrick, *Acta Crystallogr. A* **2015**, *71*, 3.

[5] G. M. Sheldrick, *Acta Crystallogr. C* **2015**, *71*, 3.

[6] C. B. Hübschle, G. M. Sheldrick, B. Dittrich, *J. Appl. Crystallogr.* **2011**, *44*, 1281.

[7] K. Fujii, Y. Ikai, H. Oka, M. Suzuki, K. Harada, *Anal. Chem.* **1997**, *69*, 5146.

[8] C. Fu, Y. Liu, C. Walt, S. Rasheed, C. D. Bader, P. Lukat, M. Neuber, F. P. J. Haeckl, W. Blankenfeldt, O. V. Kalinina et al., *Nat. Commun.* **2024**, *15*, 791.

[9] Deposition Number CCDC 2412062 ((2S,3R)-*ß*-Me-Cl-Trp) contains the supplementary crystallographic data for this paper. These data are provided free of charge by the joint Cambridge Crystallographic Data Centre and Fachinformationszentrum Karlsruhe "http://www.ccdc.cam.ac.uk/structures".

# Author Contributions

AK: isolation, complete structure elucidation, data acquisition, data analysis, writing of original draft

JFGG: fermentation, extraction, affinity chromatography, writing of original draft

SR: all biological assays, writing of original draft

MZ: acquisition and analysis of LC-MS data, writing of original draft

FF: biological assays

BM: acquisition, analysis, and writing of XRD data

RM, SZ: project planning, funding acquisition, project administration, validation
